# Compressive Remodeling Alters Fluid Transport Properties of Collagen Networks – Implications for Tumor Growth

**DOI:** 10.1038/s41598-019-50268-z

**Published:** 2019-11-20

**Authors:** J. Ferruzzi, M. Sun, A. Gkousioudi, A. Pilvar, D. Roblyer, Y. Zhang, M. H. Zaman

**Affiliations:** 1 Department of Biomedical Engineering, Boston University, Boston, MA USA; 2 Department of Mechanical Engineering, Boston University, Boston, MA USA; 3 Department of Electrical & Computer Engineering, Boston University, Boston, MA USA; 4 Howard Hughes Medical Institute, Boston University, Boston, MA USA

**Keywords:** Biophysics, Cancer, Engineering

## Abstract

Biomechanical alterations to the tumor microenvironment include accumulation of solid stresses, extracellular matrix (ECM) stiffening and increased fluid pressure in both interstitial and peri-tumoral spaces. The relationship between interstitial fluid pressurization and ECM remodeling in vascularized tumors is well characterized, while earlier biomechanical changes occurring during avascular tumor growth within the peri-tumoral ECM remain poorly understood. Type I collagen, the primary fibrous ECM constituent, bears load in tension while it buckles under compression. We hypothesized that tumor-generated compressive forces cause collagen remodeling via densification which in turn creates a barrier to convective fluid transport and may play a role in tumor progression and malignancy. To better understand this process, we characterized the structure-function relationship of collagen networks under compression both experimentally and computationally. Here we show that growth of epithelial cancers induces compressive remodeling of the ECM, documented in the literature as a TACS-2 phenotype, which represents a localized densification and tangential alignment of peri-tumoral collagen. Such compressive remodeling is caused by the unique features of collagen network mechanics, such as fiber buckling and cross-link rupture, and reduces the overall hydraulic permeability of the matrix.

## Introduction

Genomic instability is the primary driver of tumor initiation, however disease progression is increasingly recognized to be promoted by pathologic interactions between a tumor and its microenvironment, including exchange of mechanical forces with a remodeled extracellular matrix (ECM)^1,2^. Biomechanical alterations observed in tumors include accumulation of solid stresses, ECM stiffening, and interstitial fluid pressurization^3^. In particular, build-up of solid stresses within the primary tumor is caused by its growth within the host tissue^4^, but also by activation of stromal cells (e.g., cancer-associated fibroblasts) and ECM remodeling^3,5^. Remodeling, in turn, is directly related to ECM stiffening^6,7^, while fluid pressurization is due to a combination of leaky blood vessels, dysfunctional lymphatics, and a dense interstitial ECM^8^. Altered ECM structure and mechanics due to remodeling represents a distinguishing feature of desmoplastic cancers, that is cancers that contain high levels of ECM constituents, particularly hyaluronan and collagen type I. The ECM is actively remodeled by tumor and stromal cells by means of biochemical (deposition/removal) and biomechanical (tension/compression) interactions^9^. As a result, ECM remodeling involves alterations not only in content, but also spatial organization, of matrix components. In particular, accumulation of collagen in the interstitial space between cancer cells is mainly due to increased deposition^10–12^, whereas alignment of collagen occurs primarily at the tumor-stromal interface and is due to mechanical interactions with the surrounding matrix^13^. In breast cancer, collagen accumulation and alignment are powerful predictors of disease progression and survival^14,15^, and such fibrotic remodeling has been shown to induce a malignant phenotype and play a causative role in invasive behaviors^6^. Indirect evidence of mechanical engagement of collagen by primary tumors is provided by the identification of tumor-associated collagen signatures (TACS): collagen fibers have been found to be aligned parallel (TACS-2) or perpendicular (TACS-3) with respect to the tumor boundary^16^. While the presence of TACS-3 has been associated with the presence of tensional forces generated by contractile metastatic cells^13^, it can be reasoned that the presence of TACS-2 is instead associated with an earlier stage of disease in which compressive forces are applied to the ECM by uncontrolled proliferation, although little quantitative evidence has been provided so far. Physically, compression of collagen can lead to densification and stiffening without the need for de novo production. Biologically, reaction forces generated by the compressed ECM on the growing tumor can increase the metastatic potential of cancer cells^17,18^. Furthermore, compression of the ECM can affect its fluid transport properties, thus leading to fluid pressurization in the peri-tumoral stroma. While tensile ECM remodeling has received considerable attention, ECM compression needs improved biomechanical characterization and definition of how it affects tumor progression in the early stages of avascular growth. Albeit computational models have established a link between tumor growth, mechanical compression of collagen, and increased peri-tumoral interstitial fluid pressure via a reduced hydraulic permeability of the matrix^19,20^, the mechanisms underlying the structural and functional consequences of such compressive remodeling in collagen networks remain unknown. To fill this gap, here we used spheroids of epithelial breast cancer cells embedded in fibrous collagen to provide quantitative evidence of mechanical compression upon spheroid growth, and investigated the mechanical basis of compressive remodeling of collagen by combining biomechanical experiments with biophysical modeling of collagen network mechanics.

Multicellular tumor spheroids embedded in collagen represent a physiologically relevant 3D model that recapitulates key features of the tumor microenvironment, including biomechanical interactions within a 3D fibrous matrix, and biochemical gradients of nutrients and oxygen^21^. Spheroid growth has been linked with the generation of compressive stresses in agarose gels^22^, and has been implicated with the presence of TACS-2 in collagen gels^23^. However, while agarose is a cellularly inert material endowed with a linearly elastic behavior over a wide range of tensile and compressive strains, collagen I is a pathophysiologically relevant material characterized by complex mechanical properties. Typical features of collagen network mechanics include: material nonlinearity over finite strains, anisotropy due to fiber alignment along the direction of principal strain, time-dependent behavior due to inherent viscoelasticity or interstitial fluid flow, compressibility, and plasticity^24^. Collagen I monomers self-assemble *in vitro* at neutral pH and generate fibrils which further associate into networks of fibril bundles^25^, which here we will refer to as fiber networks. These networks are weakly cross-linked, thus presenting poor structural stability and mechanical function with respect to excised connective tissues. The mechanical properties of collagen gels can be significantly enhanced via cross-linking^26^, and they are commonly tested via shear rheometry^27^ and tensile testing^28^, while compressive properties are still poorly understood^29,30^. Hence, we employed multiphoton microscopy and confined compression testing – a well-established technique in the field of cartilage mechanics – to characterize the structural, mechanical, and fluid transport properties of collagen networks at various concentrations, before and after chemical cross-linking. A comparison between control and cross-linked collagen networks allowed us to separate poroelastic effects (due to interstitial fluid movements) from viscoplastic effects (due to fiber network dynamics) by modeling the experimentally observed behaviors via continuum biphasic and discrete network models. Overall, our results suggest that tumor growth induces compressive remodeling, a localized densification of collagen caused by fiber bending and cross-link rupture, which in turn reduces the hydraulic permeability of the peri-tumoral matrix.

## Results

### Compression of collagen by breast cancer spheroids

**Figure 1:**
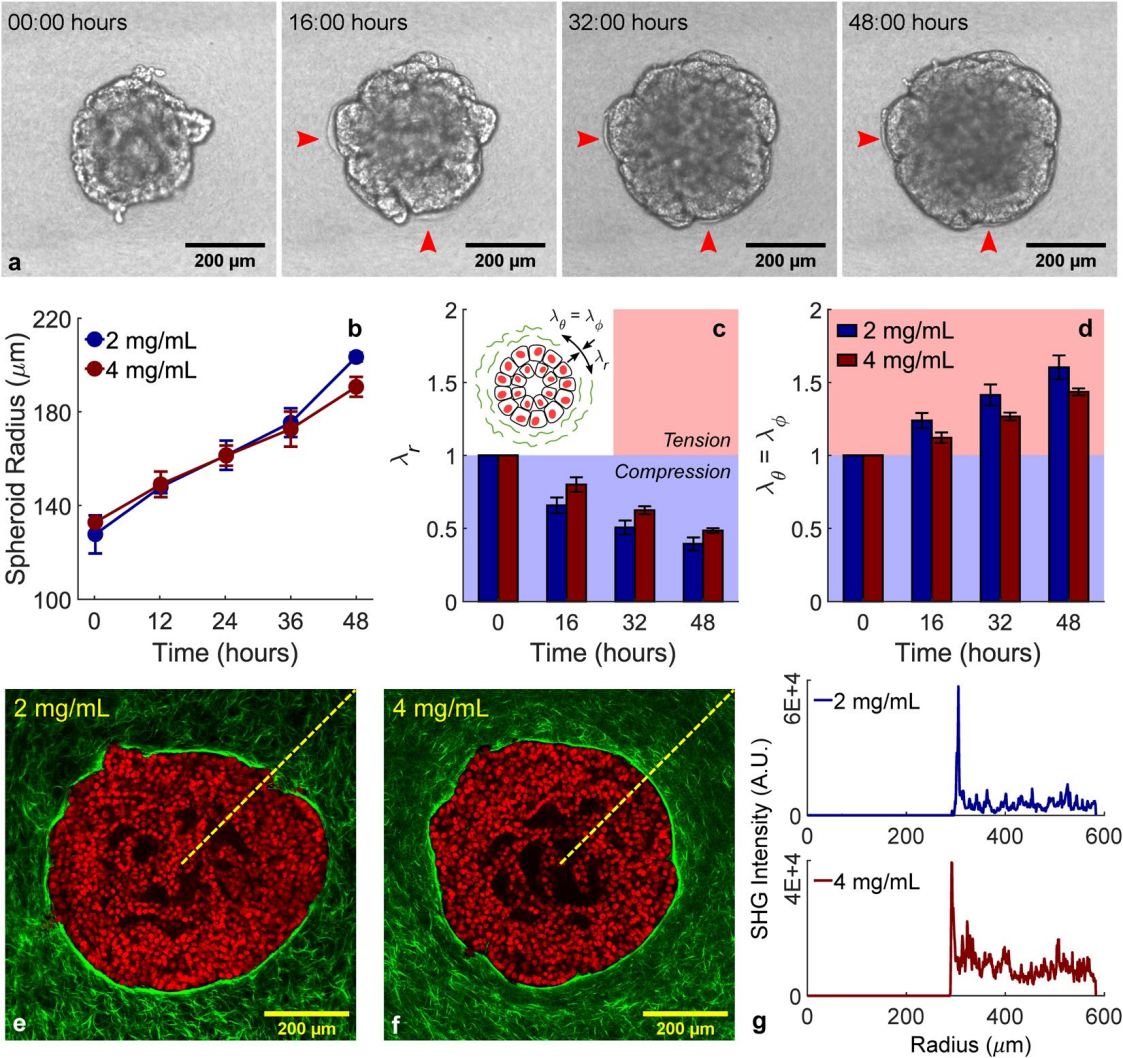
Spheroid growth induces localized radial compression in surrounding collagen. Still image frames (**a**) taken at four different time points of a representative DIC time-lapse movie of a tumor spheroid (10^3^ MCF-10A cells at the time of seeding) embedded in a 4 mg/mL collagen, with arrows indicating wrinkling of collagen due to the compressive forces generated by cell proliferation (Supplementary Video 1). Average time courses of spheroid growth in 2 and 4 mg/mL collagen (**b**) are used to estimate radial (**c**) and tangential (**d**) stretch ratios (*λ*) experienced by the surrounding matrix at the spheroid boundary as drawn schematically in the insert. Spheroids embedded in different collagen concentrations show similar growth profiles which cause radially compressive and tangentially tensile deformations. MPM images (**e,f**) display fluorescence from DAPI-stained cell nuclei (red) and SHG signal from collagen fibers (green), with the latter revealing that a dense layer of collagen accumulates at the spheroid edge as a result of its growth. Collagen densification, quantified as a peak in the radial profile of SHG intensity (**g**), indicates that compressive forces in the matrix are localized around the spheroid boundary instead of propagating in the bulk of the collagen matrix. Data are presented as mean ± SEM (n = 3 for both DIC and MPM).

Breast cancer spheroids were generated from MCF-10A human mammary epithelial cells by seeding ~10^3^ cells in low attachment wells in the presence of a small volume fraction of Matrigel (Methods). In this way, we created compacted spheroids rather than growth-arrested acinar structures^31^, which were embedded in collagen I hydrogels. MCF-10A cells possess many features of a normal mammary epithelium despite presenting genetic and epigenetic abnormalities^32^, and were therefore chosen to model an early tumor. Spheroid growth in collagen was monitored via time-lapse DIC imaging over the course of the successive 48 hours (Fig. 1a, Supplementary Video 1). Despite differences in ligand density, doubling the collagen concentration from 2 to 4 mg/mL did not significantly impact the time course of radial expansion (Fig. 1b), thus suggesting that MCF-10A spheroid growth is primarily driven by proliferation. The steady increase in spheroid radius led to visible deformations in the surrounding collagen, which estimated by modeling analytically the spheroid as an expanding spherical inclusion (Supplementary Methods). Matrix deformations, measured as stretch ratios in the principal directions $({\lambda }_{r},{\lambda }_{\theta },{\lambda }_{\varphi })$ evaluated at the spheroid-collagen boundary, were found to be radially compressive and tangentially tensile (Fig. 1c,d). More importantly, multiphoton imaging showed that such deformations do not propagate smoothly into the collagen matrix but seem to localize around the spheroid edge (Fig. 1e,f). In fact, a dense circumferential layer of collagen – which was not present immediately after embedding – is clearly evident in both collagen concentrations examined herein and reveals itself as a prominent peak in the radial profiles of SHG signal intensity (Fig. 1g), thus indicating that compressive forces localize around the spheroid-collagen boundary. Although active collagen deposition by cells may play a role in the observed densification, we hypothesized that this phenotype is caused by the mechanical forces generated by expanding tumor spheroids.

### Mechanics of collagen hydrogels under confined compression

**Figure 2:**
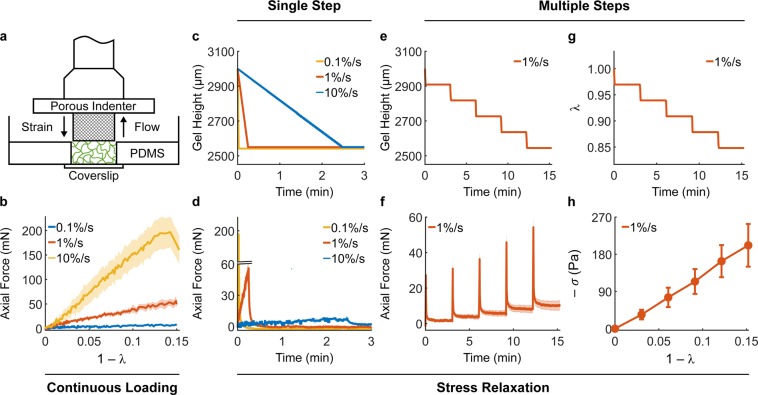
Mechanical testing under compression allows separation of fluid and solid contributions. The custom experimental set-up for confined compression (**a**) includes glass-bottom Petri dishes, PDMS wells, and a porous indenter mounted on a rheometer head (Supplementary Fig. 1). The confining PDMS well allows compressive deformations imposed on the collagen network to generate interstitial fluid flow opposite to the applied strain. Compression can be imposed via continuous loading or via stress relaxation steps. Continuous loading of 4 mg/mL collagen gels at different rates elicits significantly different mechanical responses (**b**). The same deformation and rates of compression imposed as a single stress relaxation step (**c**) shows consistent differences in peak forces but subsequent force decays to comparable values regardless of rate differences (**d**). The same absolute deformation (15%) can also be imposed via multiple steps (5 × 3%) of compression, resulting in a step-wise reduction of gel height (**e**) and axial stretch ratio *λ* (**g**). Compression of collagen gels using multiple stress relaxation steps allows us to estimate their biphasic properties. The time-course of force decay (**f** - transient response) is due to the flow of interstitial fluid pressurized upon compression while the stress at steady state (**h** - equilibrium response) is due to the mechanical properties of the solid network of collagen fibers. The material response of collagen is shown here as the compressive Cauchy stress (*−σ*) plotted against the compressive strain (1 − λ). Note how the axial force generated by collagen gels at equilibrium decays back to modest values after each step of compression. Data are presented as mean ± SEM (continuous ramp: n = 3 each, single step: n = 3 each, multiple steps: n = 4).

**Figure 3:**
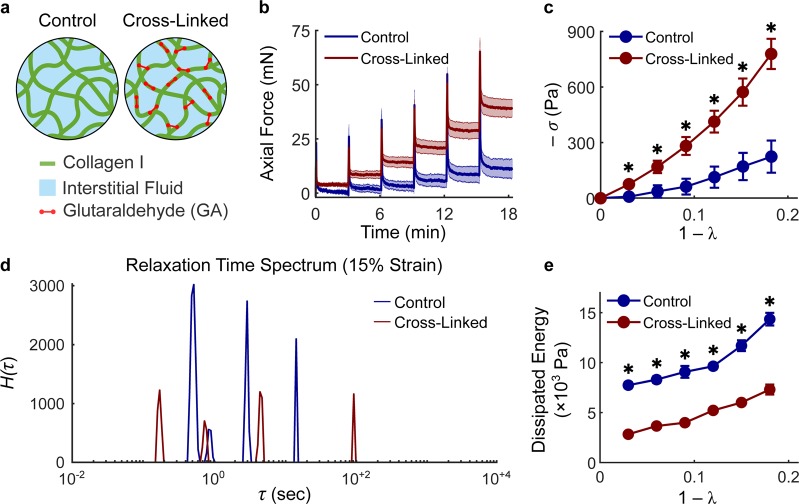
Collagen cross-linking increases hydrogel stiffness and reduces energy dissipation. At the microstructural level (**a**), a collagen gel is a network of collagen fibers (green) with pores filled by interstitial fluid (light blue), constituted in this study by either PBS or cell culture media. The collagen network can be cross-linked chemically using glutaraldehyde (GA), a bifunctional molecule (red) that strengthens both intrafibrillar and interfibrillar bonds. GA cross-linking influences both transient (**b**) and equilibrium (**c**) responses of 4 mg/mL collagen gels to confined compression. Control (blue lines) and cross-linked (red lines) gels were compressed to 18% of their original height by means of 3% steps. Cross-linked gels are characterized by a lower force decay (**b**) and a mechanical behavior that is both stiffer and nonlinearly elastic with respect to control gels (**c**). Representative relaxation time spectra *H*(*τ*) evaluated at 15% strain reveal that collagen gels display three to four distinct peaks which increase in height for increasing deformations (Supplementary Fig. 3) and are consistently lower for cross-linked, with respect to control samples (**d**). The energy dissipated at each compression step is quantified as the area under the curve of the relaxation time spectrum *H*(*τ*). Cross-linked gels display lower energy dissipation with respect to controls at all strains (**e**). Data are presented as mean ± SEM (control: n = 5, cross-linked: n = 9) and * indicates statistically significant differences with respect to controls at p < 0.05.

Collagen gels were tested mechanically under compression using a rheometer equipped with a high-resolution axial load cell and custom components, including 3D-printed self-centering parts and a porous sintered steel indenter (Supplementary Fig. 1). The resulting set-up can be easily reproduced in other laboratories. More importantly, it allowed us to impose controlled axial compression on cylindrical collagen gels, while constraining their lateral deformation via PDMS side walls, which can be considered rigid (with respect to collagen) and impermeable. External compression of the gel pressurizes the interstitial fluid, which can flow out of the collagen network through the porous indenter (Fig. 2a). Application of strains as continuous ramps at increasing rates of compression resulted, as expected, in higher axial forces (Fig. 2b) due to generation of higher interstitial fluid pressure. By holding the gel height constant after compression, one performs a stress relaxation step in which the axial force decays while the interstitial fluid flows thorough the indenter. By imposing a single step of compression, we observed that peak forces reflect the same differences observed during application of a continuous ramp but then decayed to comparable values, regardless of the compression rate (Fig. 2c,d). The equilibrium force thus reflects the underlying material properties of the solid matrix, while the time-course of force decay is caused by transient interstitial flow. Finally, compression can be imposed via multiple steps to generate a sequence of transient responses (Fig. 2e,f) that were thereupon used to characterize the fluid transport properties of collagen networks, while equilibrium responses were used to characterize their material response (Fig. 2g,h, Supplementary Methods). It should be noted that the material response of 4 mg/mL collagen gels is associated with a modest build-up of compressive stress (−*σ*) with increasing compressive strain (1 − *λ*), which is indicative of low material stiffness with respect to most soft tissues. While tissues are enzymatically cross-linked *in vivo*, collagen hydrogel are poorly cross-linked^33^. For this reason, we cross-linked collagen gels using glutaraldehyde (GA) and compared the mechanical behavior of control and cross-linked gels (Fig. 3). Preliminary tests suggested that a concentration of 0.2% v/v GA allowed us to achieve maximum functional cross-linking of collagen by strengthening both intrafibrillar and interfibrillar bonds (Fig. 3a). When exposed to similar deformations, 4 mg/mL cross-linked gels exhibited a step-wise increase in reaction forces (Fig. 3b) commonly observed when testing excised soft tissues. In addition, while peak forces were similar in magnitude between control and cross-linked gels, the force decay during the stress-relaxation phase was significantly reduced after cross-linking (Fig. 3b). Compression of collagen gels under large deformations showed that, while control gels experienced a smooth plateau of stress, cross-linked gels underwent rupture (indicated by sudden drops in force) above 20% compression (Supplementary Fig. 2). Comparison of material behavior revealed that cross-linking stiffens the gels significantly, which develop a nonlinearly elastic material behavior up to 18% deformation (Fig. 3c). The assumption of biphasic behavior implies that interstitial fluid flow is the only mechanism of energy dissipation during compression. To test this assumption, we analyzed the continuous relaxation time spectrum *H*(*τ*), which captures the distribution of time constants associated with the stress decay, at different mechanical strains for both control and cross-linked gels using an established approach^34^ (Fig. 3d). The spectra for collagen gels consisted in a series of well distinct peaks – usually 3 to 4 peaks over a wide range of relaxation times – which could reflect distinct relaxation mechanisms (Supplementary Fig. 3). The height of the peaks increased for increasing compressive strains and, regardless of strain, was lower in cross-linked samples. The area under the peaks, indicative of the energy dissipated upon compression, revealed that cross-linked networks dissipate significantly less energy with respect to controls, and in both cases energy dissipation increases for higher compressive strains (Fig. 3e). The consistent number of peaks on the relaxation time spectrum suggested us that interstitial fluid flow is not the only mechanism involved in energy dissipation upon compression, although it plays an important role in hydrogel mechanics. Overall, our results demonstrate that chemical cross-linking of collagen causes nonlinear stiffening, lower force decay and energy dissipation, as well as frank rupture at large deformations.

### Structure-function relationship of collagen networks

**Figure 4:**
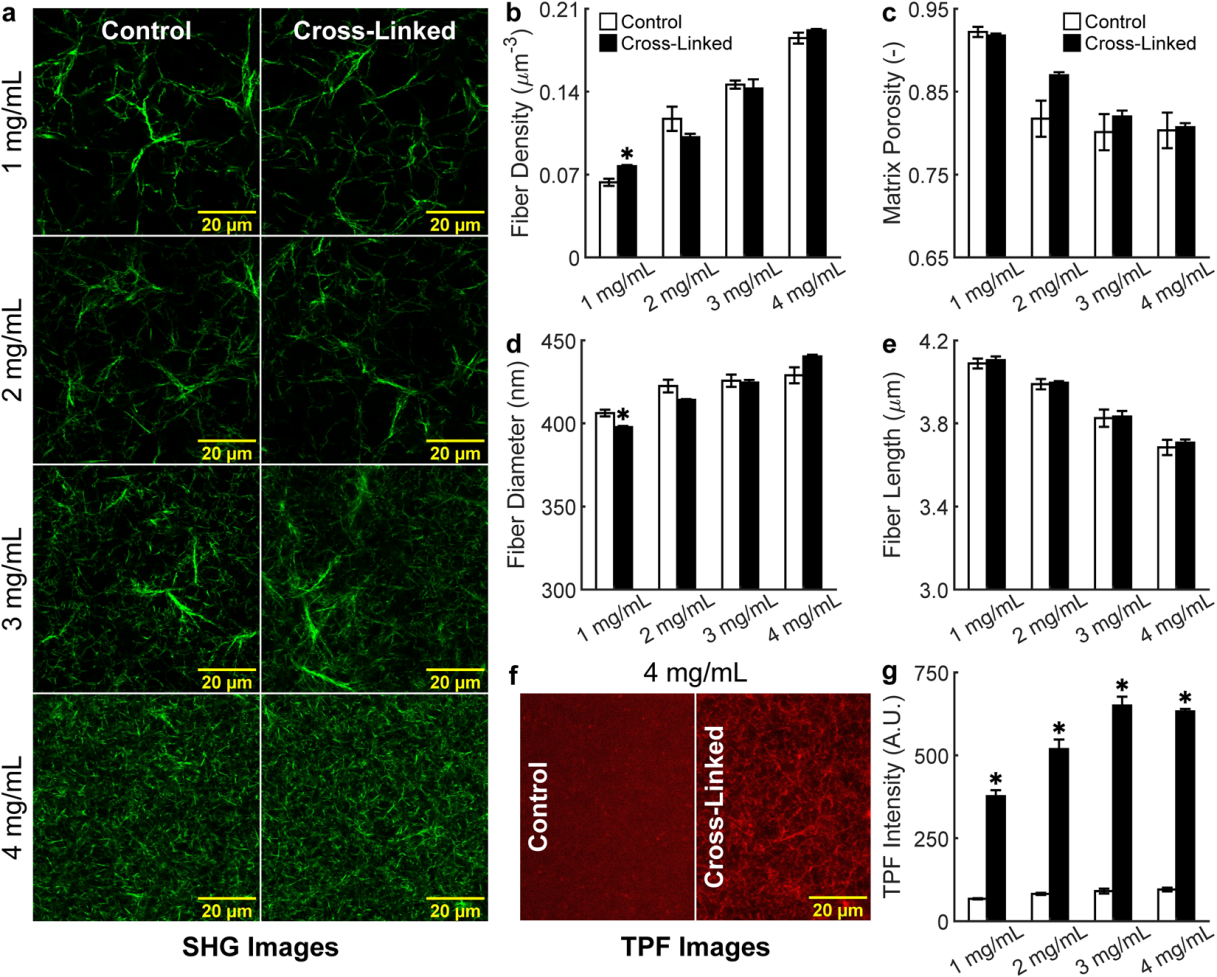
Collagen network microstructure as a function of concentration and cross-linking. MPM images were collected using 2 channels (SHG and TPF) and volumetric stacks composed of 11 images, with 3 stacks acquired at random locations for each collagen gel, 4 gels per group, 2 groups (control and cross-linked), and 4 concentrations of collagen (1, 2, 3, and 4 mg/mL), for a total of 2,112 images collected and analyzed. Representative SHG images of 1–4 mg/mL gels acquired using a 60x objective (**a**) and analyzed via global thresholding and CT-FIRE analysis (c.f., Supplementary Fig. 4) reveal that increasing collagen concentration increases dramatically fiber density (**b**) and – to a lower extent – decreases matrix porosity (**c**), while individual collagen fibers experience an increase in mean fiber diameter (**d**) and a reduction in mean fiber length (**e**). It should be noted that differences in networks structure are entirely due to concentration while little differences are due to cross-linking. Representative TPF images (**f**) show autofluorescence of unlabeled collagen cross-linked with GA upon two-photon excitation at 880 nm. In fact, mean TPF intensity (**g**) is significantly higher in cross-linked, with respect to control, gels at all concentrations. Data are shown as mean ± SEM, n = 4 per group, with numerical values and statistical analyses provided in Supplementary Table 1. * indicates statistically significant differences with respect to concentration-matched controls at p < 0.05.

**Figure 5:**
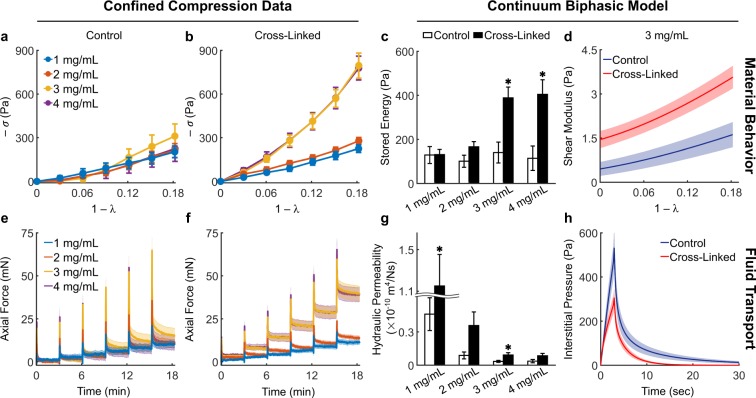
Collagen network mechanics as a function of concentration and cross-linking. Confined compression data from 1 mg/mL (control: n = 5, cross-linked: n = 9), 2 mg/mL (control: n = 6, cross-linked: n = 9), 3 mg/mL (control: n = 8, cross-linked: n = 10), and 4 mg/mL (control: n = 5, cross-linked: n = 9) gels are quantified via numerical fitting to a continuum biphasic model (Supplementary Fig. 5) to infer material behavior (top row) and fluid transport properties (bottom row) of collagen networks. Equilibrium responses from control (**a**) and cross-linked (**b**) gels compressed to 18% of their original height by means of 3% steps show that only the latter display stiffer mechanical responses for increasing collagen concentration. Increased material stiffness due to cross-linking is reflected by the higher compressive energy (**c**) stored elastically within cross-linked networks at 18% deformation and calculated from Eq. (3) as −*W*. Note how the elastic energy storage does not change with concentration for control gels. The increase in energy storage is accompanied by an increasingly nonlinear material behavior, shown here for 3 mg/mL gels as a steeper change in shear modulus with increasing strain (**d**). Transient responses from control (**e**) and cross-linked (**f**) gels show that the temporal decay in force – primarily due to flow of interstitial fluid upon compression – is affected by both collagen concentration and cross-linking. The isotropic and strain-independent permeability (**g**) estimated from experimental data decreases consistently with increasing collagen concentration due to increasing frictional losses in denser matrices. More importantly, we found that controls displayed consistently lower permeabilities with respect to cross-linked gels, despite large variability due to experimental uncertainty. Estimation of the interstitial fluid pressure (**h**) developed by 3 mg/ml gels at the bottom of the confining chamber during the first step of compression shows a higher peak pressure and a slower pressure decay in control networks. Data are shown as mean ± SEM with numerical values and statistical analyses provided in Supplementary Table 2. * indicates statistically significant differences with respect to concentration-matched controls at p < 0.05.

To gain a better understanding of the microstructural basis of macroscopic mechanics, we performed high-resolution multiphoton microscopy (MPM) imaging of control and cross-linked gels at various collagen concentrations. Second harmonic generation (SHG) images showed that, by increasing collagen concentration, the resulting networks self-assemble into more tightly packed structures (Fig. 4a). Cross-linking did not impact collagen microstructure significantly, as the gels were exposed to GA after polymerization. Quantifications via global thresholding and CT-FIRE^35^ analysis (Fig. 4b–e and Supplementary Table 1) revealed that higher collagen concentrations lead to increased fiber density and – to a lower extent – decreased matrix porosity, while individual fibers display increased diameter, decreased length, and a nearly random orientation regardless of concentration (Supplementary Fig. 4). It should be noted that the absolute values of fiber diameters are likely smaller than the values reported herein, due to the limited spatial resolution of optical microscopy. However, multiphoton imaging allowed us to quantify trends in the structure of hydrated networks (that is, without using sample altering treatments) prior to biomechanical testing. Two-photon fluorescence (TPF) images provided a functional counterpart to the structural information carried by SHG images (Fig. 4f). In fact, cross-linked networks exhibited significantly higher autofluorescence at higher concentrations and with respect to controls, thus indicating that treatment with GA altered the physical properties of individual fibers (Fig. 4g). Biomechanical properties under confined compression were separated, via biphasic modeling at the continuum scale, in material behavior and fluid transport properties (Fig. 5). Material behavior was quantified by fitting equilibrium data to a phenomenological Yeoh material model^36^. Interestingly, control gels displayed concentration-independent material properties (Fig. 5a) while cross-linked gels became stiffer at higher concentrations, with low density gels (1 and 2 mg/mL) similar to controls, while high-density gels (3 and 4 mg/mL) were significantly stiffer (Fig. 5b). The observed stiffening of high-density gels was reflected by an increase in compressive energy storage after cross-linking (Fig. 5c). We found that the shear modulus varies with strain, which indicates nonlinear properties, for both control and cross-linked gels but for the latter such response was more prominent (Fig. 5d). Fluid transport properties were quantified by fitting transient data to a biphasic mixture model (Supplementary Fig. 5). While material behavior was controlled by cross-linking, fluid transport properties were primarily controlled by concentration (Fig. 5e,f). Our results show that the hydraulic permeability exhibits a consistent decreasing trend for increasing collagen concentrations in both control and cross-linked networks (Fig. 5g). This trend in permeability is related to the underlying microstructure, with increasing frictional losses caused by interstitial fluid flow through denser matrices. More surprisingly, we found consistently lower permeabilities in control, with respect to concentration-matching cross-linked gels (Fig. 5g and Supplementary Table 2). Such trend was not reflected by our structural quantifications and revealed itself as higher peaks and slower decays in interstitial fluid pressure developed in control, with respect to cross-linked, gels (Fig. 5h and Supplementary Fig. 6). Hence, interstitial fluid flow generated under compression was found to be consistently impaired in control networks, with hydraulic permeabilities 25–40% lower with respect to their cross-linked counterparts.

### Local densification and plasticity are caused by cross-link rupture

**Figure 6:**
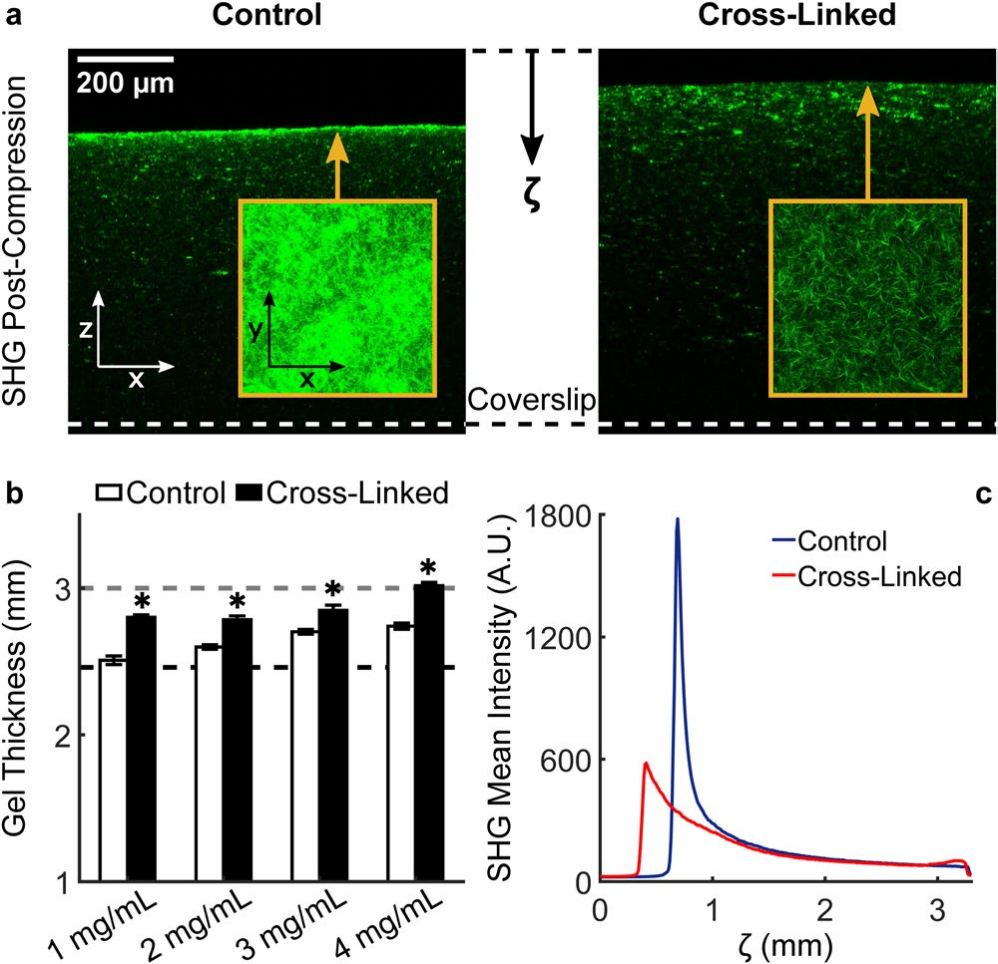
Compression induces plasticity and localized densification. After biomechanical testing, collagen gels were allowed to recover overnight at 4 °C and were subsequently imaged through their thickness (3300 µm image stacks with 10 µm steps using a 16x objective, 1x optical zoom). SHG images reveal the presence of a densified layer of collagen in control, but not in cross-linked, networks in correspondence of the gel surface that underwent compression (**a**). Representative x-z views (shrunk vertically ~4x with respect to real dimensions) show the densified layer in a representative gel. Such layer can be also visualized by taking x-y maximum intensity projections of the first 100 µm from the compressed surface (inserts). Collagen densification is associated with remnant plastic deformations in collagen gels, shown as a lower gel thickness after compression (**b**) in control, with respect to cross-linked gels, at all concentrations (1 mg/mL: n = 12, 2 mg/mL: n = 10, 3 mg/mL: n = 12, 4 mg/mL: n = 10 for both control and cross-linked gels). The gel thickness before and at the last step of compression are indicated, respectively, as grey and black dashed lines. Control and cross-linked gels at the various concentrations display various degrees of plasticity: 1 mg/ml control gels behave as perfectly plastic materials while 4 mg/ml cross-linked gels behave as perfectly elastic materials. Despite to a lower extent, even cross-linked gels at low collagen concentrations display plastic deformations. Similar to the radial decays around tumor spheroids shown in Fig. 1g, collagen densification can be visualized as a sharp peak in the SHG axial intensity profile (**c**). It should be noted that the entire thickness of the gels was imaged and that natural decay in SHG signal with depth (ζ) is due to scattering and absorption. Data are shown as mean ± SEM with numerical values and statistical analyses provided in Supplementary Table 3. * indicates statistically significant differences with respect to concentration-matched controls at p < 0.05.

**Figure 7:**
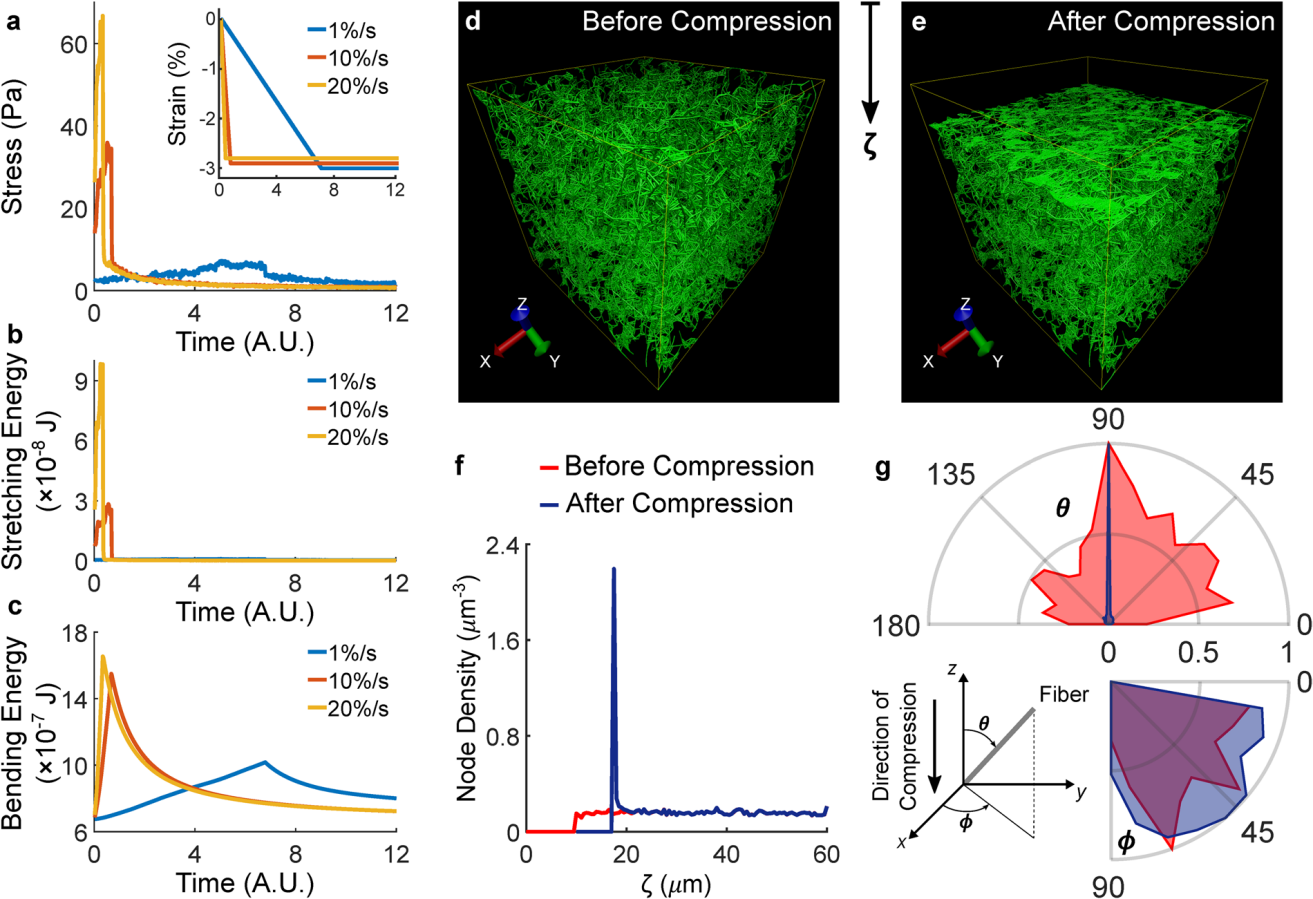
Discrete fiber network simulations capture the rate-dependent mechanical responses and localized deformations observed experimentally. The stress (**a**) developed by a simulated 4 mg/mL network compressed to 3% strain (insert) follows the stress relaxation response observed experimentally despite the peaks are less pronounced due to the lack of interstitial fluid pressurization. In this model, the stress relaxation response is due to the evolution of energy stored in stretching (**b**) and bending (**c**) upon compression. Interestingly, stretching of fibers contributes to the peak while bending of fibers dominates the decay of stress during the hold period. Comparison of network architectures before (**d**) and after (**e**) a 15% compression (Supplementary Video 2) reveals that the deformation localizes near the compressed surface, as quantified by the node density (**f**) plotted against depth (ζ) to emulate the experimental data presented in Fig. 1g and in Fig. 6c. Distributions of in-plane (*ϕ*) and out-of-plane (*θ*) fiber angles before and after compression reveal that collagen densification associates with a realignment of fibers (**g**) perpendicular to the direction of compression.

The lower hydraulic permeabilities quantified in control gels can be explained by examining the morphology of collagen hydrogels after removal of compressive loads. SHG images acquired using a long working distance objective captured the entire gel thickness and revealed the presence of a dense layer of collagen in correspondence of the gel surface that underwent compression (Fig. 6a). Collagen densification was associated with remnant plastic deformations in control gels of all concentrations, as shown by the significantly lower thicknesses after compression (Fig. 6b and Supplementary Table 3). Plasticity was also observed in low density cross-linked gels but it was less marked and not associated with localized densification of collagen. Mean SHG intensity decays with increasing depth of penetration are due to scattering and absorption, however control gels displayed a sharp peak in SHG intensity in correspondence of the dense collagen layer with subsequent decay to intensity values comparable to cross-linked gels (Fig. 6c). The densification of collagen on the surface of the gel after compression and the sharp peak in SHG axial intensity profiles were reminiscent of the densification observed around the spheroid boundary after 48 hours of proliferation (Fig. 1e–g). The presence of localized plastic deformations, along with the multiple relaxation mechanisms identified by the relaxation time spectrum analysis (Fig. 3d), clearly indicated that fluid flow is not the only mechanism underlying time-dependent responses of collagen networks during compression. Viscoplastic effects due to interactions between fibers likely underlie the localized densification of collagen upon compression identified by our experiments. The majority of continuum models used to model collagen network mechanics are purely elastic^37^ or model viscoplasticity via phenomenological relations^38^ which do not account for the physical mechanisms underlying the observed behaviors. In order to address this gap, we developed a discrete network model to gain further insights into the mechanisms of compressive matrix remodeling. A cubic 3D network of collagen fibers was generated to reproduce a 4 mg/mL gel and compressed at one of the boundaries while maintaining all the others fixed, in order to simulate the kinematics of confined compression. Individual fibers were modeled as elastic rods capable of storing elastic energy in both stretching and bending, based on their Young’s modulus *E*. Forces generated upon deformation within each fiber are transmitted through the network via cross-liking: a fiber pair exchanges stretching and bending forces through the cross-linked node until the total force acting on the cross-linked node exceeds a critical force *f*_*break*_, in correspondence of which the cross-link breaks and the two fibers move independently. Discrete fiber network simulations allowed us to reproduce qualitatively experimentally observed behaviors and gain insights into the underlying mechanisms (Fig. 7, Supplementary Video 2). In fact, rate-dependent behaviors were observed by compressing the network boundary at different speeds (Fig. 7a) and the stress decay after a step of compression was reminiscent of our experimental results (Fig. 2d). By simulating the fiber network response in absence of interstitial fluid flow, the model shows that stress relaxation in the network is due to a combination of fiber stretching and bending. The former primarily generates peak forces during compression while the latter dominates the force decay at constant deformation (Fig. 7b,c). Such decay minimizes the overall bending energy, which is one order of magnitude higher with respect to the stretching energy and represents the primary source of elastic energy storage in the network upon compression. Network architectures visualized before and after compression (Fig. 7d,e) show that fibers densify on the compressed boundary in a fashion that is remarkably similar to what we observed in control gels after compression (Fig. 6a). In fact, the sharp peak in node density after compression is similar to the peaks in SHG signals observed experimentally (Fig. 7f). The localized densification is associated with reorientation of fibers in the direction perpendicular to the applied deformation (Fig. 7g), likely caused by fiber buckling under compression.

**Figure 8:**
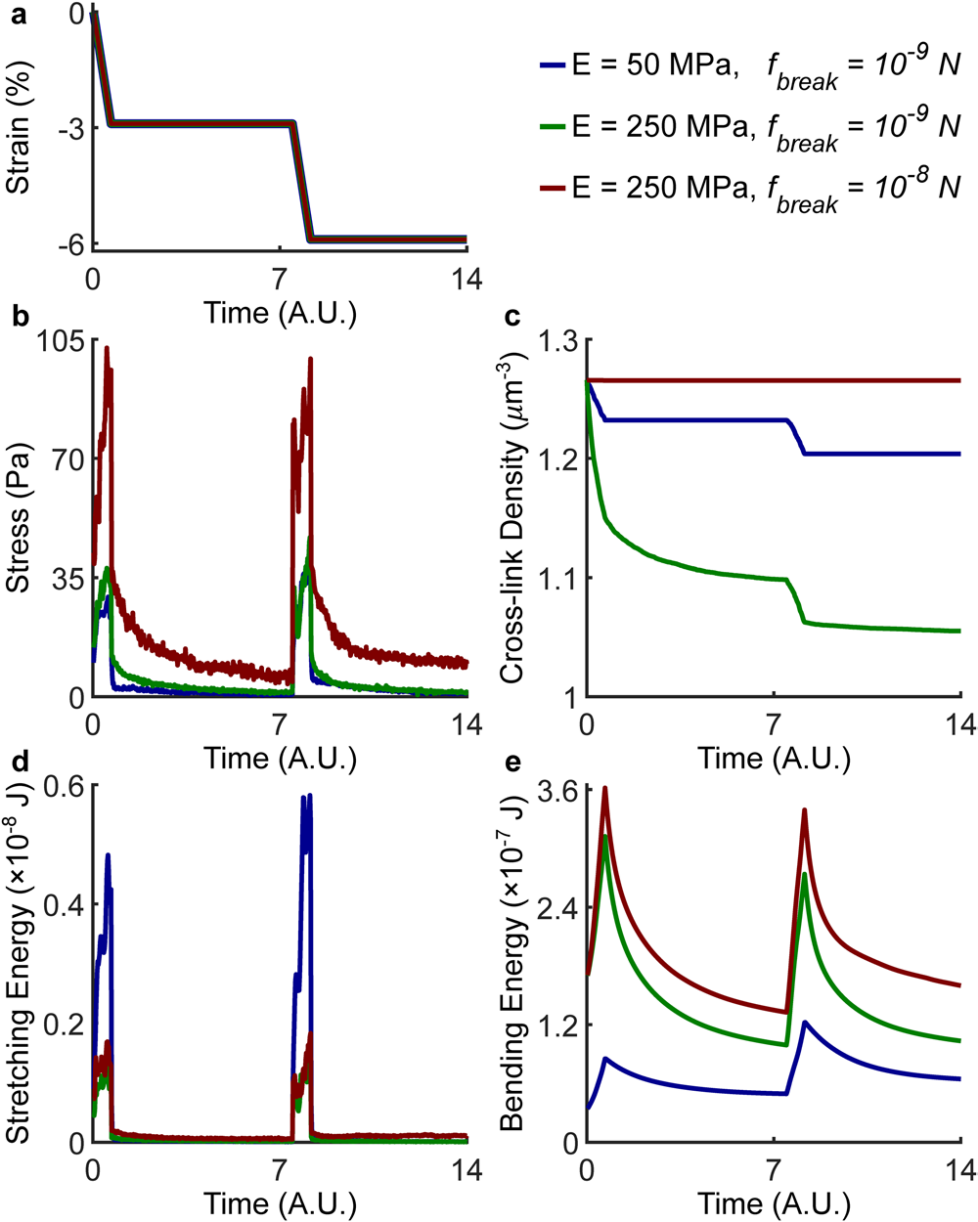
Fiber stiffness and cross-link strength govern fiber network mechanics under compression. Two consecutive steps of 3% compression (**a**) were simulated while varying the Young’s modulus *E* of individual fibers and the cross-link breaking force ${f}_{break}$ over a broad range of values. The full results of the parametric analysis are shown in Supplementary Fig. 8 while here we show the effect of changing the two key parameters on the axial stress (**b**), cross-link density (**c**), stretching energy (**d**), and bending energy (**e**). Both fiber stiffness (*E*) and cross-link strength (${f}_{break}$) need to increase in order to achieve higher equilibrium stresses, which are reminiscent of behavior observed in collagen gels cross-linked with GA. Conversely, control gels are likely characterized by lower *E* and ${f}_{break}$, thus suggesting that low bending energies and cross-link rupture are responsible for the compressive remodeling observed upon confined compression or after tumor spheroid growth.

The role played by cross-linking in the mechanics of fiber networks was explored by varying *E* and *f*_*break*_, the two key parameters that control the mechanical behavior of the network. Interestingly, the cross-link density has little impact on the simulated stress responses (Supplementary Fig. 7). While Young’s moduli of individual collagen fibrils can be measured experimentally^39^, little is known about the cross-link strength. To address this gap, we performed a parameter sweep in which *E* was varied between 10 MPa and 250 MPa while *f*_*break*_ was varied between 1 pN (10^−12^ N) and 1 µN (10^−6^ N) to cover a broad and realistic range of values. The results of such parametric study (Supplementary Fig. 8) allowed us to gain insights into the mechanical changes occurring in a fiber network after treatment with GA. Figure 8 shows simulation results for $E=50\,MPa,\,{f}_{break}={10}^{-9}\,N$ displaying negligible equilibrium stresses which are reminiscent of the responses observed in control gels. More importantly, such responses are associated with modest bending energies and rupture of cross-links at each compression step (Fig. 8). The time course of the stress was moderately affected by a 5-fold increase in the fiber Young’s modulus alone ($E=250\,MPa,\,{f}_{break}={10}^{-9}\,N$) despite the overall bending energy increased considerably. A higher fiber stiffness caused an even higher rupture of cross-links which explains the lack of stiffening at the network level. Higher peak and equilibrium stresses, similar to the responses observed in GA cross-linked gels, were observed only by increasing both *E* and ${f}_{break}$ ($E=250\,MPa,\,{f}_{break}={10}^{-8}\,N$). The stiffer responses are associated with lower stretching and higher bending energies and, more importantly, with virtually no cross-link rupture (Fig. 8c). Overall, our model shows that increasing fiber stiffness alone is not sufficient to achieve stiffer network properties, but it requires increased cross-linking strength. We conclude that both factors are affected by collagen cross-linking with GA, with stronger intrafibrillar bonds leading to higher fiber stiffness and creation of interfibrillar bonds leading to cross-linking strengthening. Conversely, our modeling results suggest that cross-link rupture and low bending energies – due to fiber buckling under compression – underlie the matrix remodeling observed in collagen hydrogels after compression and, likely, at the boundary of proliferating tumor spheroids. Such compressive remodeling alters the hydraulic permeability properties of the collagen matrix, therefore suggesting impaired convective fluid transport in the tumor microenvironment.

## Discussion

Remodeling of tissues occurs by means of biochemical activity and biomechanical forces generated by resident cells interacting with the structural and mechanical properties of the surrounding ECM. In desmoplastic cancers, ECM components can accumulate either within the interstitial space via increased production, or alternatively align along the tumor-stromal interface via expansion of the tumor mass. The existence of these distinct modalities of remodeling is confirmed by stress alleviation therapy^3^ outcomes: anti-fibrotic drugs, such as the angiotensin receptor blocker Losartan, significantly lower interstitial accumulation of ECM while leaving nearly intact the peri-tumoral fibrous capsule^10^. Such fibrous capsule, mostly made of collagen, is likely initiated in the stage of avascular growth and accumulates at the periphery as the tumor increases in size, thus generating a dense, tangentially aligned layer of collagen fibers (TACS-2). The growth-induced compressive forces responsible for peri-tumoral remodeling are also present in the interior of the tumor, where they combine with interstitial remodeling to cause interstitial fluid pressurization and intra-tumoral vessel collapse^40^. The role of interstitial ECM remodeling in the tumor interior has been documented by several studies examining the effect of ECM depletion on the physical properties of desmoplastic tumors^10–12,40^. Compared to interstitial remodeling, little work has been done to estimate peri-tumoral compressive forces in realistic environments and to characterize structural and functional changes associated with compression of the tumor microenvironment. Computational studies have shown that compression causes densification and tangential alignment of peri-tumoral collagen, while providing correlations between tumor stress, matrix permeability, and fluid velocity^19,20^. Based on the observation of similar TACS-2 phenotypes *in vivo*^16^ and *in vitro*^23^, we hypothesized that structural and functional effects of tumor-driven collagen remodeling can be isolated by employing realistic *in vitro* models, such as the multicellular spheroid embedded in a 3D collagen gel. Therefore, we created spheroids from MCF-10A cells, an epithelial cell line derived from a human mammary tumor expressing abnormal protein expression profiles^32^. We embedded MCF-10A spheroids into collagen I gels to investigate the mechanical remodeling of fibrous collagen, without confounding contributions from hyaluronan or other ECM components. Contrarily to the findings documented by Helmlinger *et al*.^22^ in agarose, varying collagen concentration does not impact significantly the time course of spheroid radial expansion (Fig. 1b), which suggests that collagen networks cannot generate enough solid stress to inhibit spheroid growth. Instead, they undergo radially compressive and tangentially tensile deformations (Fig. 1c,d) consistent with the stress distributions observed in tumors^5^. Since tumor growth induces radial compression of collagen, we refer to TACS-2 as a compressive remodeling phenotype.

We investigated the mechanisms underlying compressive remodeling of collagen via microstructural characterization, biomechanical testing, and modeling at multiple length scales. Poroelasticity represents a general mechanism regulating biomechanical properties of many soft tissues^41^, and was assumed to play an important role in collagen hydrogel mechanics, due to the elevated water content (porosities ≥80% for all collagen concentrations considered herein). The permeability of fiber networks, which regulates their fluid transport properties, is known to depend on the total volume and spatial alignment of fibers^42^ and previous studies have shown that a direct measurement of permeability (i.e., quantified from the flow rate under a known a hydrostatic pressure) is not feasible for the concentrations of collagen that are customarily used in 3D cell cultures^43^. Therefore, we used stress relaxation tests in confined compression along with a biphasic continuum model to separate material behavior from fluid transport properties. We found that, in addition to interstitial fluid flow, viscoelastic relaxation contributes to the temporal evolution of compressive reaction forces (Fig. 3) and found evidence of plastic deformations after compression (Fig. 6). In order to distinguish the contributions due to viscoplasticity, we compared control and cross-linked gel mechanics. We used glutaraldehyde (GA), a bifunctional molecule that reacts with primary amines via its aldehyde groups^44^, to form stable intrafibrillar and interfibrillar cross-links. GA cross-linking does not alter network microstructure but modifies intrinsic fiber properties, as suggested by the observed increase in collagen autofluorescence (Fig. 4). As we compared control and GA-crosslinked gels, we found that the latter behave nearly elastically, while the former displayed plastic behaviors even at small strains. As a result, concentration-dependent changes in material stiffness were evident only after cross-linking while control gels exhibited undistinguishable material properties under compression at all concentrations examined herein. Fluid transport properties are primarily controlled by collagen concentration but were found to be consistently lower in control gels due to compressive remodeling (Fig. 5g). Modeling the hydraulic permeability of collagen gels as isotropic (due to the random orientation of fibers) and strain-independent (due to the elevated porosity and relatively low strains) provided satisfactory fits to data (Supplementary Fig. 5) and no further improvements were achieved by allowing the permeability to decrease with increasing strain. In order to explore the causes of densification and plasticity, we developed a discrete network model capable of tracking the dynamic evolution of fiber networks, including transient mechanical responses and cross-link density changes during deformation. By allowing non-affine fiber kinematics, the model suggested that compressive remodeling is a result of fiber buckling and cross-link rupture. By increasing both fiber stiffness and cross-link strength, it enabled us to reproduce mechanical behaviors qualitatively similar to those observed testing GA cross-linked gels. In terms of pathophysiological relevance, collagen stiffening due to cross-linking is spatially heterogeneous and associated with the invasive front of a tumor^6,7^. Homogeneously cross-linked gels such as those used herein are not intended to be used as a model of tumor progression but, rather, as a biomechanical comparison for control gels. The latter, instead, represent a model for the normal stroma that surrounds a tumor in its early stages. Only by comparing control gels to their cross-linked counterparts we were able to identify fiber buckling and cross-link rupture as the mechanisms underlying compressive remodeling in response to tumor spheroid growth. The loss of hydraulic permeability measured with compression reflects previous findings from a model of spheroid growth in a collagenous matrix^20^. In addition to collagen, the tumor microenvironment includes hyaluronan and therefore it would be important to extend the current work by studying the effects of compressive remodeling in collagen/hyaluronan co-gels^45^, a more realistic model of the peri-tumoral stroma.

We conclude that not only overall content, but also spatial organization of collagen represents a key determinant of transport properties in fibrous networks. Compressive remodeling – that is compression-induced localized densification of collagen – is caused by fiber buckling and cross-link rupture and is associated with lower hydraulic permeability and higher interstitial fluid pressure. Our findings suggest that both solid mechanics and fluid transport properties of the tumor microenvironment can be altered by a growing tumor compressing the ECM into a fibrous capsule. In breast tumors, presence of a fibrous capsule is a common indicator of a benign lesion, despite the fact that in 10–20% of cases such well-defined tumors are carcinomas^46^. Moving forward, combined experimental and theoretical models of ECM remodeling will play a key role in providing a better understanding of how microenvironmental solid and fluid mechanics impact tumor progression.

## Methods

### Collagen and spheroid preparation

Collagen solutions were prepared by mixing high concentration, acid-solubilized rat tail collagen I (Corning Life Sciences, Bedford, MA) with equal volumes of a neutralizing buffer, consisting of a 100 mM HEPES solution in 2x PBS adjusted to pH 7.3 via addition of NaOH^47,48^. The desired collagen concentrations (1–4 mg/mL) were reached by adding adequate volumes of neutralized collagen to cold 1x PBS. Collagen solutions were prepared on ice and allowed to self-assemble into networks inside an incubator (37 °C) for 1 hour. Acellular gels for biomechanical testing were formed by aliquoting 200 µL of liquid collagen inside cylindrical PDMS molds with a diameter of 9 mm (Supplementary Methods). Cross-linking was achieved by adding glutaraldehyde (GA) diluted in deionized water (dH_2_O) to acellular gels. For each collagen concentration, gels were randomly divided into two groups after polymerization: one was incubated with 4 mL of dH_2_O (control) while the other was exposed to the same volume of 0.2% v/v GA (Sigma-Aldrich, St. Loius, MO) at room temperature for 12 hours (cross-linked). Control and cross-linked gels were then washed-out twice using 2 mL of 1x PBS. Multicellular spheroids were cultured and embedded in collagen as follows. MCF-10A cells (ATCC, Manassas, VA) were cultured in DMEM/F-12 supplemented with 5% horse serum, 20 ng/mL EGF, 0.5 mg/mL hydrocortisone, 100 ng/mL cholera toxin, 10 μg/mL insulin, and 1% penicillin/streptomycin. Cells were maintained at 37 °C and 5% CO_2_ and, before reaching confluence, were trypsinized, counted, and resuspended at a concentration of 10^6^ cells/mL. Spheroids were generated by seeding ~10^3^ cells per well in a 96-well ultra-low attachment plate and allowed to form for 48 hours in presence of 2.5% v/v Matrigel^49^. Once formed, spheroids were transferred individually into glass bottom 6-well plates (MatTek, Ashland, MA) and embedded within 150 μL of liquid collagen at concentrations of 2 and 4 mg/mL. Collagen self-assembled within an incubator for 1 hour and the cell culture plates were carefully rotated every minute for the first 10 minutes in order to guarantee that the spheroids were fully embedded within the collagen matrix and were not in touch with the glass coverslip.

### Time-lapse imaging and analysis

Spheroid growth in collagen was imaged using a customized spinning disk confocal setup consisting of a Leica DMI 6000B microscope (Leica, Wetzlar, Germany) equipped with an ImagEM CCD camera (Hamamatsu Photonics, Hamamatsu, Japan), a FW-1000 high speed filter wheel (Applied Scientific Instrumentation, Eugene, OR), and a LiveCell environmental chamber (Pathology Devices, San Diego, CA). 2 mL of culture media were added to each well and the plate was maintained at 37 °C, 5% CO_2_, and 80% humidity for the entire duration of the imaging study. For each spheroid within a 6-well plate, differential interference contrast (DIC) images were collected every 10 minutes for 48 hours as 3 × 3 tiled, 200 μm z-stacks. A 10x objective was used to image an area of 1450.3 × 1450.3 μm^2^ with a resolution of 1.126 μm/pixel. Image acquisition was controlled using the MicroManager 1.4 software (https://micro-manager.org). Minimum intensity projections were used to visualize the 3D data sets as 2D movies and to ensure that the spheroid equator was captured in subsequent analyses. The spheroid radius at each time frame was measured using a modified version of an established algorithm^50^. Briefly, the center of the spheroid was found by minimization of the Mumford-Shah functional while determination of the spheroid radius was carried out by calculating the spherically symmetric image “graininess” – a first-order approximation to the square of the image gradient^50^ – and by determining the furthest point where the radial graininess drops below 75% of its maximum. The distance between such point and the center represents the spheroid radius, which approximates well the spheroid size under the assumption of spherical symmetry.

### Multiphoton microscopy and analysis

After 48 hours of DIC imaging, spheroids embedded in collagen were fixed overnight using cold 4% PFA and stained using 2 μM DAPI. Tumor spheroids and fibrous collagen were imaged using a Bruker Ultima Investigator multiphoton microscope (MPM). A laser beam (Insight DeepSee+, Spectra Physics, Santa Clara, CA) with an excitation wavelength of 880 nm was focused onto the samples through either a 16x water objective (Nikon, 0.8 N.A., WD = 3 mm) or a 60x oil objective (Olympus, 1.42 N.A., WD = 0.15 mm). Two-photon fluorescence (TPF) from DAPI stained nuclei and second harmonic generation (SHG) signal from the collagen matrix were collected, respectively, using 550/50 nm and 440/40 nm bandpass filters. Spheroids were imaged using a 16x objective (1x optical zoom) and the following settings: 1024 × 1024 pixels at a resolution of 0.805 µm/pixel, a stack size of 600 µm with 5 µm steps. Matrix densification upon spheroid growth was visualized via radial profiles of SHG intensity. Similarly, plastic remodeling of acellular gels after biomechanical testing was visualized via axial profiles of SHG intensity. The overall gel thickness was measured as the distance between the gel surface and the glass coverslip, and for each sample we report the mean thickness from three measurements at separate x-y locations. Microstructural features of acellular collagen networks were imaged using a 60x objective (2x optical zoom) and the following settings: 1024 × 1024 pixels at a resolution of 0.076 µm/pixel, a stack size of 10 µm with 1 µm steps. High-resolution imaging of microstructural details was consistently carried out 5 µm away from the coverslip to avoid artifacts. Image stacks were thresholded and the mask from each SHG image was applied to the corresponding TPF image, which varies in intensity as a function of collagen cross-linking^51^. Following Raub *et al*.^51^, the threshold was defined as the mean plus twice the standard deviation of the maximum SHG intensity measured in a small area containing no discernible collagen fibers. The largest threshold among all experimental groups was used as the global SHG intensity threshold and used to generate a mask for each image. These SHG masks were then used to segment collagen fibers and quantify mean TPF intensity of collagen as well as mean fiber area. Due to the random orientation of collagen fibers, the volumetric porosity equals the areal porosity^52^ which was quantified directly from the masks. Finally, mean fiber structure was extracted using CT-FIRE, a validated algorithm for segmentation of microscopy images and extraction of fiber geometry and alignment^35^. Analysis of images with CT-FIRE led to quantification of fiber orientation (deg), diameter (nm), length (µm), and density (µm^−3^).

### Biomechanical testing

Custom components (Supplementary Fig. 1) were developed to perform confined compression tests on acellular collagen gels using a commercial DHR-2 rheometer (TA Instruments, New Castle, DE). A precompression of ~144 µm was applied to cylindrical gels to bring them to a thickness *H* = 3 mm, which was thereupon regarded as the unloaded thickness. Contact between the indenter and the hydrogel surface was confirmed by a spike in the axial force (*f*) registered by the rheometer. The baseline force (*f*_*off*_) was used to offset the axial force experimentally measured (*f*^exp^) during compression. Unless otherwise stated, compression steps of 3% were applied at a constant rate of 1%/s and followed by hold periods of 180 s, during which the deformation remained constant while the force decreased to reach a plateau. Experimental data on gap height and axial force were sampled at a frequency of 100 Hz. The deformed gel thickness *h* was obtained subtracting the glass coverslip thickness to the value of gap height controlled by the rheometer. The axial stretch ratio was thus calculated as $\lambda =h/H$. The axial stress was instead calculated as

1 $$-{\sigma }^{\exp }=\frac{{f}^{\exp }-{f}_{off}}{\pi {D}^{2}/4},$$

where *D* = 8 mm represents the diameter of the porous indenter. Herein we follow the convention that the Cauchy stress (*σ*) is positive under tension and negative under compression. Equilibrium values of stretch and stress were calculated based on the average of the last 60 seconds in each hold phase. Data analysis and numerical modeling were carried out using Matlab R2018a (Mathworks, Natick, MA), unless otherwise specified. Stress relaxation data were analyzed using a generalized Maxwell model, in which the time evolution of the Cauchy stress is described as

2 $$\sigma (t)={\sigma }_{e}+{\int }_{-\infty }^{+\infty }H(\tau ){e}^{-t/\tau }d\,\mathrm{ln}\,\tau ,$$

where *H*(*τ*) is the continuous relaxation time distribution function and *τ* represents the relaxation time. A solution for *H*(*τ*) was found by solving a minimization problem (Supplementary Methods). The relaxation time was varied from 10^−2^ to 10^4^ seconds, for both control and cross-linked gels. For each sample, the area under the entire spectrum at each compression step was calculated using the Matlab function *trapz()*.

### Continuum biphasic modeling

Collagen hydrogels represent mixtures consisting of a solid phase (collagen fibers) and a fluid phase (interstitial liquid). Based on the observed expulsion of interstitial fluid during compression, we implemented a biphasic model of collagen hydrogel mechanics – rooted in the theory for biphasic mixtures developed in the field of cartilage mechanics^53,54^ – to fit experimental data from confined compression. The observed bulk mechanical behavior under compression was separated into a deformation-dependent stress generated by the solid collagenous matrix and a transient pressurization generated by the fluid filling the interstitial space (Supplementary Methods). Briefly, the material behavior of the solid constituent was described using the Yeoh strain energy function^36^

3 $$W={c}_{1}({I}_{C}-3)+{c}_{2}{({I}_{C}-3)}^{2}+{c}_{3}{({I}_{C}-3)}^{3},$$

where ${I}_{C}=tr\,{\bf{C}}$ represents the first invariant of the Cauchy-Green tensor, while *c*_1_, *c*_2_, and *c*_3_ are material parameters subjected to the constraints ${c}_{1},{c}_{3} > 0$ and ${c}_{2} < 0$. The fluid transport properties of collagen hydrogels were instead described by a hydraulic permeability tensor **k**, which – according to Darcy’s law – regulates hydraulic flow in porous media in response to pressure gradients^52^. Here, we assumed an isotropic and strain-independent hydraulic permeability, that is

4 $${\bf{k}}=k{\bf{I}},$$

this assumption is due to the fact that collagen gels are highly porous materials made of randomly oriented fibers. Based on the assumed constitutive behaviors, the theoretically-calculated axial stress in the mixture was indicated as ${\sigma }_{ZZ}(Z,t)$ (Supplementary Methods), where *Z* is the position of a material point along the gel height (with *Z* = 0 at the interface with the porous indenter and *Z* = *H* at the bottom of the confining chamber) while *t* represents time. The unknown model parameters were determined via nonlinear least squares minimization of the following objective function

5 $$e=\mathop{\sum }\limits_{i=1}^{N}{[{\sigma }_{ZZ}(0,t)-{\sigma }^{\exp }(t)]}_{i}^{2},$$

where ${\sigma }^{\exp }(t)$ represents the experimentally-measured Cauchy stress from Eq. (1), *N* represents the number of experimental data points, from multiple steps of compression, that were included in the regression. The objective function was minimized using the Matlab function *lsqnonlin()*, which was used within a two-step fitting procedure^55^: material parameters from the Yeoh model (*c*_1_, *c*_2_, *c*_3_) were determined by fitting equilibrium data, while the hydraulic permeability (*k*) was determined by fitting transient responses.

### Discrete network modeling

The mechanisms underlying compressive remodeling of collagen gels after compression were investigated computationally using a discrete network model. The dynamic evolution of a 3D network of collagen fibers was simulated using the framework originally developed by Kim *et al*.^56^ to model cytoskeletal networks, with the main difference being that herein we disregarded Brownian forces due to the athermal nature of collagen mechanics^57,58^. Briefly, collagen fibers with a fixed diameter of 155 nm^39,59^, a length sampled from an experimentally measured distribution (Supplementary Fig. 4), and random orientations were generated within a 50 × 50 × 50 µm^3^ cubic volume to achieve a concentration of 4 mg/mL (Supplementary Methods). Each fiber was discretized using small segments of length ${l}_{0}=1\,{\rm{\mu }}{\rm{m}}$ which, being shorter than the persistence length of collagen ${l}_{p}=5\mbox{--}10\,{\rm{\mu }}{\rm{m}}$^59^, could therefore be assumed to behave like linear elastic rods. The mechanical behavior of individual fiber segments was assumed to be governed by stretching and bending potentials which are characterized by the following equations

6 $$\begin{array}{c}{U}_{s}=\frac{1}{2}{k}_{s}{(l-{l}_{0})}^{2},\\ {U}_{b}=\frac{1}{2}{k}_{b}{(\theta -{\theta }_{0})}^{2},\end{array}$$

where *l* and *l*_0_ represent the current and equilibrium lengths of an individual fiber segment, while *θ* and *θ*_0_ represent the current and equilibrium angles formed between two adjacent segments. The stretching and bending stiffness constants are, respectively, given by ${k}_{s}=EA/{l}_{0}$ and ${k}_{b}=EI/{l}_{0}$, where *E* is the Young’s modulus of a collagen fiber, $A=\pi {R}^{2}$ is the cross-sectional area, and $I=\pi {R}^{4}/4$ is the second moment of inertia of a fiber of radius *R*. Rigid cross-links were formed between adjacent fibers and the cross-link density was varied by changing the maximum distance between fibers allowed to form a cross-link (Supplementary Methods). Using this approach, the forces developed upon deformation in one of the fibers that are part of a rigid cross-link are transferred to the other fiber until the total force at a cross-link exceeds a breaking force ${f}_{break}$, in correspondence of which the covalent bond ceases to exist and the two fibers are free to move independently. The parameters *E* and ${f}_{break}$ characterize the mechanical behavior of the fiber network and were therefore varied parametrically to reproduce qualitatively the various behaviors observed experimentally. The Young’s modulus of collagen fibrils is reported to be on the order of hundreds of MPa^39^, hence we varied *E* between 10 MPa and 250 MPa. On the other hand, little is known about the cross-link breaking force which was therefore varied between 1 pN (10^−12^ N) and 1 µN (10^−6^ N) to cover a broad range of values. It should be noted that this range was chosen to include the force needed to break intrafibrillar bonds between collagen monomers^60^. Discrete fiber network simulations were implemented in Matlab and visualized using Visual Molecular Dynamics (VMD, https://www.ks.uiuc.edu/Research/vmd).

### Statistical analysis

Experimental data are presented as mean ± standard error of the mean (SEM), based on fixed or variable sample sizes depending on the type of experiment. Due to the unequal sample sizes, differences between control and cross-linked samples were determined using a Welch’s t-test, which assumes unequal variances. A one-way ANOVA was used to test differences due to collagen concentration, and post-hoc pairwise comparisons were performed using the Bonferroni correction. Differences were considered significant for p < 0.05.

## Supplementary information

Supplementary information

Supplementary Video 1

Supplementary Video 2

## References

[1] Spill F, Reynolds DS, Kamm RD, Zaman MH (2016). Impact of the physical microenvironment on tumor progression and metastasis. Current Opinion in Biotechnology.

[2] Kai F, Drain AP, Weaver VM (2019). The Extracellular Matrix Modulates the Metastatic Journey. Developmental Cell.

[3] Stylianopoulos T, Munn LL, Jain RK (2018). Reengineering the Physical Microenvironment of Tumors to Improve Drug Delivery and Efficacy: From Mathematical Modeling to Bench to Bedside. Trends in Cancer.

[4] Nia HT (2016). Solid stress and elastic energy as measures of tumour mechanopathology. Nature Biomedical Engineering.

[5] Stylianopoulos T (2013). Coevolution of Solid Stress and Interstitial Fluid Pressure in Tumors During Progression: Implications for Vascular Collapse. Cancer Res.

[6] Levental KR (2009). Matrix Crosslinking Forces Tumor Progression by Enhancing Integrin Signaling. Cell.

[7] Acerbi I (2015). Human breast cancer invasion and aggression correlates with ECM stiffening and immune cell infiltration. Integrative. Biology.

[8] Heldin C-H, Rubin K, Pietras K, Östman A (2004). High interstitial fluid pressure — an obstacle in cancer therapy. Nat Rev Cancer.

[9] Conklin MW, Keely PJ (2012). Why the stroma matters in breast cancer: Insights into breast cancer patient outcomes through the examination of stromal biomarkers. Cell Adhesion &. Migration.

[10] Diop-Frimpong B, Chauhan VP, Krane S, Boucher Y, Jain RK (2011). Losartan inhibits collagen I synthesis and improves the distribution and efficacy of nanotherapeutics in tumors. Proceedings of the National Academy of Sciences.

[11] Chauhan, V. P. *et al*. Angiotensin inhibition enhances drug delivery and potentiates chemotherapy by decompressing tumour blood vessels. *Nature Communications* **4** (2013). 10.1038/ncomms3516 PMC3806395 24084631

[12] Papageorgis P (2017). Tranilast-induced stress alleviation in solid tumors improves the efficacy of chemo- and nanotherapeutics in a size-independent manner. Scientific Reports.

[13] Provenzano PP, Inman DR, Eliceiri KW, Trier SM, Keely PJ (2008). Contact Guidance Mediated Three-Dimensional Cell Migration is Regulated by Rho/ROCK-Dependent Matrix Reorganization. Biophysical Journal.

[14] Provenzano, P. P. *et al*. Collagen density promotes mammary tumor initiation and progression. *BMC Medicine* **6** (2008). 10.1186/1741-7015-6-11 PMC2386807 18442412

[15] Conklin MW (2011). Aligned Collagen Is a Prognostic Signature for Survival in Human Breast Carcinoma. The American Journal of Pathology.

[16] Provenzano, P. P. *et al*. Collagen reorganization at the tumor-stromal interface facilitates local invasion. *BMC Medicine* **4** (2006). 10.1186/1741-7015-4-38 PMC1781458 17190588

[17] Demou ZN (2010). Gene Expression Profiles in 3D Tumor Analogs Indicate Compressive Strain Differentially Enhances Metastatic Potential. Annals of Biomedical Engineering.

[18] Tse JM (2012). Mechanical compression drives cancer cells toward invasive phenotype. Proceedings of the National Academy of Sciences.

[19] Wijeratne PA (2016). Multiscale modelling of solid tumour growth: the effect of collagen micromechanics. Biomechanics and Modeling in Mechanobiology.

[20] Wijeratne PA, Hipwell JH, Hawkes DJ, Stylianopoulos T, Vavourakis V (2017). Multiscale biphasic modelling of peritumoural collagen microstructure: The effect of tumour growth on permeability and fluid flow. PloS one.

[21] Charoen KM, Fallica B, Colson YL, Zaman MH, Grinstaff MW (2014). Embedded multicellular spheroids as a biomimetic 3D cancer model for evaluating drug and drug-device combinations. Biomaterials.

[22] Helmlinger G, Netti PA, Lichtenbeld HC, Melder RJ, Jain RK (1997). Solid stress inhibits the growth of multicellular tumor spheroids. Nature Biotechnology.

[23] Carey SP, Starchenko A, McGregor AL, Reinhart-King CA (2013). Leading malignant cells initiate collective epithelial cell invasion in a three-dimensional heterotypic tumor spheroid model. Clinical & Experimental Metastasis.

[24] Ferruzzi J., Zhang Y., Roblyer D., Zaman M. H. (2019). Multi-scale Mechanics of Collagen Networks: Biomechanical Basis of Matrix Remodeling in Cancer. Multi-scale Extracellular Matrix Mechanics and Mechanobiology.

[25] Christiansen DL, Huang EK, Silver FH (2000). Assembly of type I collagen: fusion of fibril subunits and the influence of fibril diameter on mechanical properties. Matrix Biology.

[26] Hapach LA, VanderBurgh JA, Miller JP, Reinhart-King CA (2015). Manipulation of *in vitro* collagen matrix architecture for scaffolds of improved physiological relevance. Physical Biology.

[27] Janmey PA (2007). Negative normal stress in semiflexible biopolymer gels. Nature Materials.

[28] Roeder BA, Kokini K, Sturgis JE, Robinson JP, Voytik-Harbin SL (2002). Tensile Mechanical Properties of Three-Dimensional Type I Collagen Extracellular Matrices With Varied Microstructure. J Biomech Eng.

[29] Chandran PL, Barocas VH (2004). Microstructural Mechanics of Collagen Gels in Confined Compression: Poroelasticity, Viscoelasticity, and Collapse. Journal of Biomechanical Engineering.

[30] Kim OV (2017). Compression-induced structural and mechanical changes of fibrin-collagen composites. Matrix Biology.

[31] Debnath J, Muthuswamy SK, Brugge JS (2003). Morphogenesis and oncogenesis of MCF-10A mammary epithelial acini grown in three-dimensional basement membrane cultures. Methods.

[32] Soule HD (1990). Isolation and characterization of a spontaneously immortalized human breast epithelial cell line, MCF-10. Cancer research.

[33] Veis Arthur, George Anne (1994). Fundamentals of Interstitial Collagen Self-Assembly. Extracellular Matrix Assembly and Structure.

[34] Wang Y, Li H, Zhang Y (2018). Understanding the viscoelastic behavior of arterial elastin in glucose via relaxation time distribution spectrum. Journal of the Mechanical Behavior of Biomedical Materials.

[35] Bredfeldt JS (2014). Computational segmentation of collagen fibers from second-harmonic generation images of breast cancer. Journal of Biomedical Optics.

[36] Yeoh OH (1990). Characterization of Elastic Properties of Carbon-Black-Filled Rubber Vulcanizates. Rubber Chemistry and Technology.

[37] Ban Ehsan, Wang Hailong, Franklin J. Matthew, Liphardt Jan T., Janmey Paul A., Shenoy Vivek B. (2019). Strong triaxial coupling and anomalous Poisson effect in collagen networks. Proceedings of the National Academy of Sciences.

[38] Ban E (2018). Mechanisms of Plastic Deformation in Collagen Networks Induced by Cellular Forces. Biophysical Journal.

[39] van der Rijt JAJ, van der Werf KO, Bennink ML, Dijkstra PJ, Feijen J (2006). Micromechanical Testing of Individual Collagen Fibrils. Macromolecular Bioscience.

[40] Martin JD (2019). Dexamethasone Increases Cisplatin-Loaded Nanocarrier Delivery and Efficacy in Metastatic Breast Cancer by Normalizing the Tumor Microenvironment. ACS Nano.

[41] Ehret, A. E. *et al*. Inverse poroelasticity as a fundamental mechanism in biomechanics and mechanobiology. *Nature Communications* **8** (2017). 10.1038/s41467-017-00801-3 PMC5714996 29042539

[42] Stylianopoulos T (2008). Permeability calculations in three-dimensional isotropic and oriented fiber networks. Physics of Fluids.

[43] Ramanujan S (2002). Diffusion and Convection in Collagen Gels: Implications for Transport in the Tumor Interstitium. Biophysical Journal.

[44] Olde Damink LHH (1995). Glutaraldehyde as a crosslinking agent for collagen-based biomaterials. Journal of Materials Science: Materials in Medicine.

[45] Lai VK (2016). Swelling of Collagen-Hyaluronic Acid Co-Gels: An *In Vitro* Residual Stress Model. Ann Biomed Eng.

[46] Yoo JL (2010). Can MR Imaging Contribute in Characterizing Well-circumscribed Breast Carcinomas?. RadioGraphics.

[47] Sung KE (2009). Control of 3-dimensional collagen matrix polymerization for reproducible human mammary fibroblast cell culture in microfluidic devices. Biomaterials.

[48] Harjanto D, Maffei JS, Zaman MH (2011). Quantitative Analysis of the Effect of Cancer Invasiveness and Collagen Concentration on 3D Matrix Remodeling. PLoS ONE.

[49] Ivascu A, Kubbies M (2006). Rapid Generation of Single-Tumor Spheroids for High-Throughput Cell Function and Toxicity Analysis. Journal of Biomolecular Screening.

[50] Stein AM (2007). Estimating the cell density and invasive radius of three-dimensional glioblastoma tumor spheroids grown *in vitro*. Applied Optics.

[51] Raub CB (2007). Noninvasive Assessment of Collagen Gel Microstructure and Mechanics Using Multiphoton Microscopy. Biophysical Journal.

[52] DULLIEN F.A.L. (1979). Pore Structure. Porous Media.

[53] Mow VC, Kuei SC, Lai WM, Armstrong CG (1980). Biphasic Creep and Stress Relaxation of Articular Cartilage in Compression: Theory and Experiments. J Biomech Eng.

[54] Holmes MH (1986). Finite Deformation of Soft Tissue: Analysis of a Mixture Model in Uni-Axial Compression. J Biomech Eng.

[55] Ateshian GA, Warden WH, Kim JJ, Grelsamer RP, Mow VC (1997). Finite deformation biphasic material properties of bovine articular cartilage from confined compression experiments. Journal of Biomechanics.

[56] Kim T, Hwang W, Kamm RD (2009). Computational Analysis of a Cross-linked Actin-like Network. Experimental Mechanics.

[57] Stein AM, Vader DA, Weitz DA, Sander LM (2011). The micromechanics of three-dimensional collagen-I gels. Complexity.

[58] Licup AJ (2015). Stress controls the mechanics of collagen networks. Proceedings of the National Academy of Sciences.

[59] Sivakumar L, Agarwal G (2010). The influence of discoidin domain receptor 2 on the persistence length of collagen type I fibers. Biomaterials.

[60] Depalle B, Qin Z, Shefelbine SJ, Buehler MJ (2016). Large Deformation Mechanisms, Plasticity, and Failure of an Individual Collagen Fibril with Different Mineral Content. Journal of Bone and Mineral Research.

