# Supplementary material for: Compressive Remodeling Alters Fluid Transport Properties of Collagen Networks – Implications for Tumor Growth
Source: Sci Rep. 2019 Nov 20;9:17151. doi: 10.1038/s41598-019-50268-z (PMC6868165; doi:10.1038/s41598-019-50268-z)
Supplement: Supplementary file 1 — Supplementary information [file 41598_2019_50268_MOESM1_ESM.pdf]

Supplementary Information

Compressive Remodeling Alters Fluid Transport Properties of Collagen Networks – Implications for Tumor Growth

J. Ferruzzi[1*], M. Sun[1], A. Gkousioudi[2], A. Pilvar[3], D. Roblyer[1], Y. Zhang[2], M.H. Zaman[1,4*]

[1]Department of Biomedical Engineering
Boston University, Boston, MA, USA

[2]Department of Mechanical Engineering
Boston University, Boston, MA, USA

[3]Department of Electrical & Computer Engineering
Boston University, Boston, MA, USA

[4]Howard Hughes Medical Institute
Boston University, Boston, MA, USA

*Correspondence and requests for materials should be addressed to J.F. (jacopofe@bu.edu) or to M.H.Z. (zaman@bu.edu).

SUPPLEMENTARY METHODS

Estimation of collagen deformation upon spheroid growth. The deformation gradient tensor $\mathbf{F} = diag\left(\lambda_r, \lambda_\theta, \lambda_\phi\right)$ – where λ_i represents the stretch ratio in the principal direction i, expressed using spherical coordinates – was calculated to estimate the state of deformation imposed on collagen by growing spheroids following the approach outlined by Bower[1]. Briefly, the collagen matrix was regarded as a spherically symmetric, hollow solid with internal radius R_0, equal to the initial spheroid radius, and external radius R_1. For the purpose of numerical evaluation, R_1 was assumed to be at least one order of magnitude larger than R_0. At each time point, the internal radius r_0 corresponds to the measured spheroid radius, whereas the external radius is assumed to not be impacted by the expanding internal radius, and thus is maintained constant. Using a spherical-polar coordinate system, a point within the undeformed collagen matrix is described by coordinates (R,Θ,Φ), while in any deformed state is described by coordinates (r,θ,ϕ). Assuming quasi-static motions, the kinematics can be described as

$$r = r(R), \quad \theta = \Theta, \quad \phi = \Phi, \tag{S1}$$

that is, each material point moves only radially due to the spherical symmetry albeit it is stretched in all three principal directions. In fact, the deformation gradient assumes diagonal form $\mathbf{F} = diag\left(\partial r/\partial R, r/R, r/R\right)$ and the Jacobian is given by $J = \det \mathbf{F} = \left(R_1^3 - r_0^3\right)/\left(R_1^3 - R_0^3\right)$. The principal stretches, that is the non-zero components of $\mathbf{F}$, can be calculated as a function of the undeformed radius R via integration

$$\begin{aligned} \lambda_r\left(R\right) &= JR^2\left[r_0^3 + J\left(R^3 - R_0^3\right)\right]^{-2/3}, \\ \lambda_\theta\left(R\right) = \lambda_\phi\left(R\right) &= \frac{1}{R}\left[r_0^3 + J\left(R^3 - R_0^3\right)\right]^{-1/3}, \end{aligned} \tag{S2}$$

from which the principal stretches at the spheroid-matrix boundary ($R = r_0$) imposed by cellular proliferation can be evaluated and are reported in Figure 1.

Custom components for biomechanical testing. Cylindrical wells made of polydimethylsiloxane (PDMS) with a diameter of 9 mm were created inside 35 mm glass-bottom petri dishes (MatTek Corporation, Ashland, MA) by using 3D printed self-centering cylinders (Supplementary Fig. 1a). Briefly, 1 mL of a liquid PDMS mixture (Dow Corning, Midland, MI) with a 10:1 weight ratio of silicon elastomer to curing agent, was deposited inside the dishes and around the cylinders, and cured at 50°C for 2 hours (Supplementary Fig. 1b). After removal of the cylinder, the resulting cylindrical well was cleaned from PDMS residues and coated using poly-D-lysine (Sigma-Aldrich, St. Loius, MO) and GA, thus providing an anchoring layer for collagen to avoid gel floating. After rinsing with 1x PBS and sterilizing under ultraviolet (UV) light, cylindrical gels were obtained by polymerizing 200 μL of liquid collagen inside each PDMS well (Supplementary Fig. 1c). Next, the petri dishes were positioned in a DHR-2 rheometer (TA Instruments, New Castle, DE) by using a 3D printed adaptor (Supplementary Fig. 1d) to ensure that each collagen gel was centered with respect to the rotational axis of the instrument. By locking the rotation and by controlling the gap distance, the DHR-2 rheometer was used to record the axial loads generated by collagen gels upon compression, thanks to an axial load cell with a resolution of 0.5 mN. The confining PDMS well did not allow lateral deformations or fluid flow, thus we used a porous indenter to compress collagen while allowing interstitial fluid flow in the axial direction. Disks made of a 316L sintered steel mesh with pores in the range of 4-100 μm (Porvair Filtration Group, Ashland, VA) were cut to a diameter of 8 mm via wire electrical discharge machining to ensure that the pores on all surfaces would remain open. The resulting porous cylinder was centered and press-fitted on a 25 mm rheometer plate to allow mounting on the DHR-

2 stage (Supplementary Fig. 1D-E). In addition to accurate control of deformations and measurement of forces, this experimental set-up allowed for a quick and reliable centering of collagen gels with respect to the porous indenter, while leaving a lateral 500 µm clearance to avoid interference with the PDMS walls.

Relaxation time spectrum analysis. Most biomaterials exhibit viscoelastic behavior which can be modeled as a linear combination of springs and dashpots. A generalized Maxwell model is a mechanical model consisting of many Maxwell elements in parallel. In its most general form[2], the number of Maxwell elements approaches infinity and the time evolution of the Cauchy stress is described as

$$\sigma(t) = \sigma_e + \int_{-\infty}^{+\infty} H(\tau) e^{-t/\tau} d\ln\tau, \tag{S3}$$

where $H(\tau)$ is the continuous relaxation time distribution function and τ represents the relaxation time. Equation (S3) has the form of a Fredholm integral equation of the first kind and solving it for $H(\tau)$ is considered an ill-posed problem because the solution is highly sensitive to experimental noise. Tikhonov regularization was used to solve equation (S3) by introducing additional constraints to the solution $H(\tau)$ via a regularization parameter, Λ. According to this method, the function $H(\tau)$ is obtained by minimizing the quantity

$$V(\lambda) = \sum_{i=1}^{N} \left[Z_i - \left(\sigma_e + \int_{-\infty}^{+\infty} H(\tau) e^{-t/\tau} d\ln\tau \right) \right]^2 + \Lambda \left\| LH(\tau) \right\|^2, \tag{S4}$$

where N is the number of experimental data points, Z is the experimental Cauchy stress during the stress relaxation phase and $\|\bullet\|$ represents the Euclidean norm of the matrix. The operator L is the second derivative with the constraint that $H(\tau)$ vanishes smoothly at the upper or lower boundary of the time interval[3]. The regularization parameter controls the weight given to the second term,

the Euclidean norm of the solution, in order to be small. With an appropriate value of Λ, the first term forces the results to be compatible with the data, while the second term leads to a smooth estimate of the solution $H(\tau)$ [4]. Herein, the minimization problem was solved using the FTIKREG software in which the solution method is based on the generalized singular-value decomposition[4]. For each sample, we analyzed the temporal evolution of the Cauchy stress during the stress relaxation phase of each compression step, which consisted of ~9800 experimental data points.

Continuum biphasic model formulation. We implemented a finite deformation theory for biphasic mixtures[5–8] to fit experimental data from confined compression experiments and isolate the contributions of solid stress and fluid pressurization to the observed bulk properties. The average mechanical behavior of the mixture is weighed based on the volume fraction of each constituent, defined as $\varphi^\alpha = V^\alpha / V$, where $\alpha = s,f$ indicates solid and fluid constituents, V^α and V represent constituent and mixture volumes respectively, and the biphasic mixture is assumed to be fully saturated ($V^s + V^f = V$, or $\varphi^s + \varphi^f = 1$). Each constituent is assumed to be intrinsically incompressible (i.e., it preserves its volume) while the mixture can undergo volumetric changes during compression. The fluid is treated as inviscid (it can only support a pressure, not a shear stress) because frictional interactions within the fluid are considered negligible with respect to those between solid and fluid, which represent the primary source of energy loss. Due to the experimentally observed nonlinear responses, the solid constituent is treated as hyperelastic, and its mechanical response to deformation depends on a free energy density function *W*. Therefore, the Cauchy stress in individual constituents can be written as follows

$$\begin{aligned} \boldsymbol{\sigma}^f &= -\varphi^f p\mathbf{I}, \\ \boldsymbol{\sigma}^s &= -\varphi^s p\mathbf{I} + \boldsymbol{\sigma}^E, \end{aligned} \tag{S5}$$

where bold quantities represent second-order tensors. In particular, $\mathbf{I}$ is the identity tensor, $\boldsymbol{\sigma}^{\alpha}$ represents the Cauchy stress tensor in constituent α. The stress in the mixture is calculated as $\boldsymbol{\sigma} = \boldsymbol{\sigma}^{f} + \boldsymbol{\sigma}^{s}$. The interstitial fluid pressure p contributes to the overall Cauchy stress via an isotropic stress given by $-p\mathbf{I}$, while the solid matrix contributes via a deformation-dependent (or extra) Cauchy stress $\boldsymbol{\sigma}^{E}$, which can be calculated as

$$\boldsymbol{\sigma}^{E} = \frac{2}{J}\mathbf{F}\frac{\partial W}{\partial \mathbf{C}}\mathbf{F}^{T}, \tag{S6}$$

where $\mathbf{F}$ is the deformation gradient tensor and $J = \det \mathbf{F}$ is its Jacobian (which reflects changes of volume), while $\mathbf{C} = \mathbf{F}^{T}\mathbf{F}$ is the right Cauchy-Green tensor. Experimentally, the mixture deformation is described by a deformation gradient of the form $\mathbf{F} = \mathrm{diag}(1, 1, \lambda)$, where $\lambda = h/H$ represents the axial stretch controlled via the rheometer. It should be noted that in confined compression $h < H$, and thus $J < 1$, which confirms that the mixture loses volume due to expulsion of interstitial fluid. Constitutive equations are needed to describe the linear momentum exchange between the solid and fluid phase and the material behavior of the solid phase. Following Ateshian[9], the linear momentum exchange is governed by the hydraulic permeability tensor $\mathbf{k}$, which was assumed to be isotropic and strain-independent, that is

$$\mathbf{k} = k\mathbf{I}, \tag{S7}$$

This assumption was further supported by the fact that implementation of a strain-dependent permeability[8,10] did not improve the quality of data fitting while increasing the number of unknown model parameters (results not shown). Regarding the material behavior of the solid phase, we employed the phenomenological strain energy function originally developed by Yeoh[11] to describe rubber elasticity

$$W = c_1(I_C - 3) + c_2(I_C - 3)^2 + c_3(I_C - 3)^3 + \frac{1}{D}(J - 1)^2, \tag{S8}$$

where $I_C = tr\mathbf{C}$ represents the first invariant of the Cauchy-Green tensor, while c_1, c_2, and c_3 are material parameters subjected to the constraints $c_1, c_3 > 0$ and $c_2 < 0$. The Yeoh material model was chosen because it represents the simplest strain energy function for which the shear modulus, instead of being constant, varies with deformation and thus reproduces the nonlinear material behavior observed in collagen gels under compression. In fact, other functional forms – including the neo-Hookean and Mooney-Rivlin models – did not describe well the material behavior from our experiments (results not shown). It should be noted that, with respect to equation (3), equation (S8) includes a volumetric part (last term) which depends on the inverse of the bulk modulus *D* and on the Jacobian *J*. The additive decomposition of *W* into isochoric and volumetric parts allowed us to account for the decrease in mixture volume during confined compression, and to enforce theoretically the incompressibility of the solid phase. In fact, proper differentiation of equation (S8) followed by imposition of $J = 1$, leads to the following form of the extra Cauchy stress

$$\boldsymbol{\sigma}^E = \left[2c_1 + 4c_2\left(I_C - 3\right) + 6c_3\left(I_C - 3\right)^2\right]\left(\mathbf{B} - \frac{1}{3}I_C\mathbf{I}\right), \tag{S9}$$

where $\mathbf{B} = \mathbf{F}\mathbf{F}^T$ is the left Cauchy-Green tensor. By substituting equations (S7) and (S9) into the linear momentum balance for the biphasic mixture, one obtains the following governing equation

$$\frac{\partial U}{\partial t} = \frac{16}{3}k\left[\frac{9}{2}c_3\left(1 + \frac{\partial U}{\partial Z}\right)^4 + \left(2c_2 - 9c_3\right)\left(1 + \frac{\partial U}{\partial Z}\right)^2 + c_1 - 4c_2 + 9c_3\right]\frac{\partial^2 U}{\partial Z^2}, \tag{S10}$$

where $U(Z,t)$ represents the Lagrangian displacement generated within the collagen hydrogel during compression, while Z is the position of a material point along the gel height with $Z = 0$ at the interface with the porous indenter and $Z = H$ at the bottom of the confining chamber. Due to axi-symmetry of both gel geometry and external loads, the displacement field depends only on the

axial position Z. Equation (S10) represents a nonlinear parabolic PDE, which was solved numerically under the following Dirichlet initial and boundary conditions

$$U(Z,0)=0,$$
$$U(0,t)=\begin{cases} V_0 t & for \quad 0\leq t<t_0 \\ V_0 t_0 & for \quad t_0\leq t<t_f \end{cases}, \tag{S11}$$
$$U(H,t)=0,$$

where the displacement prescribed experimentally by the indenter is separated into a phase of compression at rate V_0 for a period t_0 and a phase in which the imposed compression is held constant until the end of the compression step ($t = t_f$). Equation (S10) was discretized using an implicit finite difference formulation and solved numerically via Newton's method to obtain the displacement field $U(Z,t)$. Following Holmes[6], from the calculated displacement field one can compute the strain field as $\lambda(Z,t)=1+\partial U/\partial Z$, the axial component of the extra stress $\sigma_{ZZ}^{E}(Z,t)$ from equation (S9), and the interstitial fluid pressure as $p(Z,t)=\sigma_{ZZ}^{E}(Z,t)-\sigma_{ZZ}^{E}(0,t)$. The last result derives from imposing mechanical equilibrium on the mixture[6] and ensures that the pressure at the interface with the porous indenter ($Z=0$) is constantly zero because the interstitial fluid is in equilibrium with the surrounding atmospheric pressure. Finally, the axial stress field in the mixture is calculated as

$$\sigma_{ZZ}(Z,t)=-p(Z,t)+\sigma_{ZZ}^{E}(Z,t), \tag{S12}$$

Network model implementation. A 3D fiber network was generated computationally within a 50×50×50 μm^3 domain to model the mechanical behavior of a 4 mg/mL collagen gel undergoing confined compression by using the parameters summarized in Supplementary Table 4. Fiber length, diameter, orientation, density, mechanical properties, and cross-linking between fibers were assigned according to the following rules. A gamma distribution

$$PDF(l) = b^{-a} l^{a-1} e^{-l/b} / \Gamma(a), \tag{S13}$$

was used to fit the distribution of fiber length extracted using CT-FIRE[12,13] (Supplementary Fig. 4) so that experimental variations could be captured via the parameters a (shape) and b (scale). Fiber diameters, instead, were not measured directly because microscopy methods (including SHG) are not capable of resolving fiber diameters that are smaller than the diffraction limit for optical wavelengths. Therefore the fiber diameter was assumed to be constant and was fixed at $d = 155\ \text{nm}$ [14,15]. Each fiber was seeded using a random number generator within the simulation domain, and the fiber subsequently extended along a direction randomly selected from uniform distributions of polar and azimuthal angles until a final contour length, randomly sampled from $PDF(l)$, was reached. The total number of fibers was determined to achieve a concentration of 4 mg/mL, which was computed using the volume fraction of fibers and the true mass density of collagen[16], that is $\rho_T^c = 1.35\ \text{g/cm}^3$. Fiber were discretized using segments of length $l_0 = 1\ \mu\text{m}$ which, being shorter than the persistence length of collagen $l_p = 5 - 10\ \mu\text{m}$ [15], could therefore be assumed to behave like linear elastic rods. Each end of a segment represents a network node which is capable of forming cross-links with nodes from other fibers. After network generation, nodes from distinct collagen fibers that were separated by a distance lower or equal to the cross-link distance l_{xlink} were assumed to form rigid cross-links. The impact of cross-link density on the simulated network mechanics was evaluated by varying l_{xlink} parametrically between 1 and 8 µm (Supplementary Fig. 7 and Supplementary Table 4). Rigid and free-slip boundaries were assigned to all sides of the cubic volume and, in order to reproduce the deformations generated during a confined compression experiment, one of the sides was displaced inwardly at a rate that was varied between 1%/s and 20%/s, to simulate rate dependent responses. Once the network reached a

compression equal to 3%, the moving boundary was thereupon held still. The stress generated by the network at the compression boundary was calculated by first adding up all the reaction forces of fiber nodes within 1 μm of distance from the boundary, and then dividing the normal component (with respect to the boundary) of the total reaction force by the area of the compression boundary (50×50 μm^2). The dynamic evolution of the network was simulated by enforcing mechanical equilibrium at each node via Newton's second law[17,18]

$$m_i \frac{d^2\mathbf{x}_i}{dt^2} = \mathbf{f}_i - \zeta_i \left(\frac{d\mathbf{x}_i}{dt} - v_\infty \right), \tag{S14}$$

where m_i represents the mass of a collagen segment, $\mathbf{x}_i$ is the *i*-th node position, $\mathbf{f}_i$ is the resultant of all the external forces acting on the *i*-th node, v_∞ represents the velocity of the surrounding fluid, and ζ_i is the friction coefficient acting on a cylindrical fiber of diameter *d* which, depending on the direction, can be approximated as[18]

$$\begin{aligned} \zeta_{i,\perp} &= 3\pi\eta d \frac{\left(3 + 2 l_0/d\right)}{5}, \\ \zeta_{i,\parallel} &= 3\pi\eta d \frac{\left(4 + l_0/d\right)}{5}, \end{aligned} \tag{S15}$$

where η is the viscosity of the surrounding fluid (Supplementary Table 4). The friction coefficients in the transverse ($\zeta_{i,\perp}$) and parallel ($\zeta_{i,\parallel}$) directions were used to dampen fiber motion upon bending and stretching, respectively. Equation (S14) was simplified by assuming that inertial terms are negligible at the length and time scales of interest, in addition to assuming negligible flow ($v_\infty = 0$) of the surrounding fluid[18]. The network was thus driven out of its mechanical equilibrium by imposing a compressive step displacement at one of its boundaries, and the subsequent position of each node was updated by solving the simplified equation (S14) via a fourth-order Runge-Kutta scheme. Individual fiber segments were assumed to behave as linear

elastic rods, hence their response to applied deformations is characterized by the following functional forms

$$U_s = \frac{1}{2} k_s \left(l - l_0\right)^2 ,$$
$$U_b = \frac{1}{2} k_b \left(\theta - \theta_0\right)^2 , \tag{S16}$$

where l and l_0 represent the current and equilibrium distances between two adjacent nodes along a fiber, while θ and θ_0 represent the current and equilibrium angles formed between two adjacent segments. The stretching and bending stiffness constants are, respectively, given by $k_s = EA/l_0$ and $k_b = EI/l_0$, where $A = \pi R^2$ is the cross-sectional area and $I = \pi R^4/4$ is the second moment of inertia of a fiber of radius R. Therefore, the Young's modulus E governs both stretching and bending behavior of an individual fiber. An additional force acting on a node is provided by rigid cross-linking. That is, if two nodes i and j from distinct fibers are bound by a cross-link, they experience a force given by

$$\mathbf{f}_{i,j} = \mathbf{f}_i^s + \mathbf{f}_i^b + \mathbf{f}_j^s + \mathbf{f}_j^b \quad if \quad \left|\mathbf{f}_{i,j}\right| < f_{break} , \tag{S17}$$

where $\mathbf{f}_i^s$ and $\mathbf{f}_i^b$ represent forces generated on node i via stretching and bending of the first fiber, while $\mathbf{f}_j^s$ and $\mathbf{f}_j^b$ represent forces generated on node j via stretching and bending of the second fiber. That is, the forces acting on each node are transferred rigidly through the cross-link until the total force $\mathbf{f}_{i,j}$ exceeds the breaking force f_{break}, in correspondence of which the covalent bond ceases to exist and the two nodes are free to move independently.

SUPPLEMENTARY DISCUSSION

Collagen gels are routinely tested under conditions of tensile[19–21] or shear[22–24] loading, which engage the collagen fibers directly in tension. Compression testing is often used to extract bulk properties from collagen networks regardless of its relevance to the conditions of interest, mainly because of its relatively simple experimental set-up[25–27]. Roy et al.[25] performed stress-relaxation experiments on glycated collagen networks and interpreted the resulting data using a mono-exponential linear viscoelastic model, which resulted in the quantification of an equilibrium modulus and a time constant for various concentrations of ribose. Other than allowing consistent comparisons between different experimental conditions, such measures give little insight into the behavior of collagen networks under compression. More informative experiments were conducted by Kim et al.[28,29] who compressed collagen, fibrin, and collagen-fibrin composites using a commercial rheometer paired with a confocal microscope. The stress-strain response was linear under small strains, then underwent sudden softening and reached a plateau spanning a broad range of compressive strains. Direct visualization of fiber deformations showed that such plateau was associated with fiber buckling, which thus led to compaction of the network with little resistance to the imposed deformation. They also documented the presence of a "compaction front"[30], that is a layer of densified matrix near the surface of the piston which resembles the plastically remodeled layer observed in the present study (Figure 6). At high deformations, the stress-strain behavior becomes highly nonlinear due to extreme densification of the network, which was also confirmed by our experiments (Supplementary Fig. 2). Other investigators have found the behavior of collagen to be nonlinear also at low strains, and such nonlinearity is influenced by varying the rate of compression, collagen density, and degree of cross-linking[31,32]. In addition, collagen gels as a whole are found to be highly compressible with the loss of volume that has been described using

a nonlinear Poisson's ratio[32] or a coupling term between the principal stretches[33]. In particular, Ban et al.[33] recently explored the compressibility of collagen networks under multiaxial deformations and acknowledged the need to account also for the contribution of interstitial fluid pressurization and flow through the network pores to the overall mechanical properties. This type of mechanical behavior is typical of biphasic materials and can be described using Biot's theory of poroelasticity[34] or Truesdall's mixture theory[35].

Collagen hydrogels are biphasic mixtures made of collagen fibers (solid) and interstitial fluid (liquid) which are not physically bound but instead can undergo relative motions upon mechanical loading[9]. Such biphasic structure results in time-dependent poroelastic[36] and highly compressible[37] behaviors, despite individual constituents are known to be incompressible[38]. The individual contribution of solid and liquid constituents to the overall mechanical response under compression can be separated by using confined, rather than unconfined, compression. Confined compression experiments on collagen hydrogels have proven to be difficult to conduct and interpret[36,39–41]. Following the theoretical framework originally developed by Mow and colleagues for articular cartilage[42], the first confined compression tests aimed at characterizing the biphasic properties of collagen gels were first carried out by Barocas and colleagues[39–41]. Among other findings, Chandran and Barocas[39] showed that different mechanical and structural responses can be induced in stress-relaxation tests by varying the rate of compression: a slow compression (ramp test) was associated with more uniform deformations and stresses that were maintained for longer times, while a fast compression (step test) was instead associated with network collapse near the piston and higher stresses which decayed rapidly over time. Overall, they found that collagen fibers deform heterogeneously upon compression by aligning perpendicular to the direction of applied strain, storing energy primarily via bending[39]. More recently, Busby et al.[36] found that increasing

collagen concentration increases the linear elastic modulus while decreasing the hydraulic permeability, as a result of a reduced porosity. Our work confirms and extends previous finding while introducing a consistent experimental-computational framework at multiple length scales that can now be used to characterize collagen remodeling and tumor-matrix interactions. We characterized the structure-function relationship of collagen gels, quantified material properties and hydraulic permeabilities by using a continuum biphasic framework combined with a nonlinear constitutive model, showed structural rearrangements and localized densification of control gels after compression, and proposed a mechanism for compressive remodeling informed by fiber network simulations. Our network model has the benefit of tracking the dynamic evolution of fiber network mechanics under compression, including transient mechanical responses and cross-link density changes with time during a stress relaxation test, as opposed to modeling approaches that simulate only equilibrium responses[33,43,44]. In addition to these advantages, our approach includes some limitations, including the elevated number of parameters and the need for tuning some of the physical parameters in order to achieve realistic simulation times (Supplementary Table 4). In particular, we found that the choice of simulation time step dt depends on the value of fluid viscosity η that appears in equations (S15) and effectively dampens the motion of fibers within the network. By increasing the value of η, one can increase dt and achieve realistic computational times while retaining qualitatively similar mechanical responses in the fiber network. The exact combination of dt and η dictates the time scale of the network response to the imposed compressive deformation and, in order to avoid misinterpretations, the time axes of Figures 7, 8, S7, and S8 were expressed in arbitrary units (A.U.). We used simulated responses from the network model that qualitatively match our experimental results to gain insights into the mechanisms of compressive remodeling instead of quantitatively comparing them to experimental data, which can

be done using a continuum-level descriptor of collagen mechanics. Despite these limitations, the discrete network model allowed us to reproduce the dynamical response of fiber networks under external compression, explore the sources of localized densification and plasticity, and determine the source of mechanical stiffening upon cross-linking with glutaraldehyde. Both our modeling and experimental efforts have focused only on uniaxial compression, but can be extended to other and more complex deformations modes in future studies.

REFERENCES

1. Bower, A. F. *Applied Mechanics of Solids*. (CRC Press, 2009).
2. Ferry, J. D. *Viscoelastic Properties of Polymers, 3rd Edition*. (Wiley, 1980).
3. Wang, Y., Li, H. & Zhang, Y. Understanding the viscoelastic behavior of arterial elastin in glucose via relaxation time distribution spectrum. *Journal of the Mechanical Behavior of Biomedical Materials* **77**, 634–641 (2018).
4. Weese, J. A reliable and fast method for the solution of Fredhol integral equations of the first kind based on Tikhonov regularization. *Computer Physics Communications* **69**, 99–111 (1992).
5. Holmes, M. H., Lai, W. M. & Mow, V. C. Singular Perturbation Analysis of the Nonlinear, Flow-Dependent Compressive Stress Relaxation Behavior of Articular Cartilage. *J Biomech Eng* **107**, 206–218 (1985).
6. Holmes, M. H. Finite Deformation of Soft Tissue: Analysis of a Mixture Model in Uni-Axial Compression. *J Biomech Eng* **108**, 372–381 (1986).
7. Kwan, M. K., Lai, W. M. & Mow, V. C. A finite deformation theory for cartilage and other soft hydrated connective tissues—I. Equilibrium results. *Journal of Biomechanics* **23**, 145–155 (1990).
8. Holmes, M. H. & Mow, V. C. The nonlinear characteristics of soft gels and hydrated connective tissues in ultrafiltration. *Journal of Biomechanics* **23**, 1145–1156 (1990).
9. Ateshian, G. A. Mixture Theory for Modeling Biological Tissues: Illustrations from Articular Cartilage. in *Biomechanics: Trends in Modeling and Simulation* (eds. Holzapfel, G. A. & Ogden, R. W.) 1–51 (Springer International Publishing, 2017). doi:10.1007/978-3-319-41475-1_1

10. Lai, W. M. & Mow, V. C. Drag-induced compression of articular cartilage during a permeation experiment. *Biorheology* **17**, 111–123 (1980).

11. Yeoh, O. H. Characterization of Elastic Properties of Carbon-Black-Filled Rubber Vulcanizates. *Rubber Chemistry and Technology* **63**, 792–805 (1990).

12. Bredfeldt, J. S. *et al.* Computational segmentation of collagen fibers from second-harmonic generation images of breast cancer. *Journal of Biomedical Optics* **19**, 016007 (2014).

13. Liu, Y., Keikhosravi, A., Mehta, G. S., Drifka, C. R. & Eliceiri, K. W. Methods for Quantifying Fibrillar Collagen Alignment. in *Fibrosis* (ed. Rittié, L.) **1627**, 429–451 (Springer New York, 2017).

14. van der Rijt, J. A. J., van der Werf, K. O., Bennink, M. L., Dijkstra, P. J. & Feijen, J. Micromechanical Testing of Individual Collagen Fibrils. *Macromolecular Bioscience* **6**, 697–702 (2006).

15. Sivakumar, L. & Agarwal, G. The influence of discoidin domain receptor 2 on the persistence length of collagen type I fibers. *Biomaterials* **31**, 4802–4808 (2010).

16. Cusack, S. & Miller, A. Determination of the elastic constants of collagen by Brillouin light scattering. *Journal of Molecular Biology* **135**, 39–51 (1979).

17. Kim, T., Hwang, W. & Kamm, R. D. Computational Analysis of a Cross-linked Actin-like Network. *Experimental Mechanics* **49**, 91–104 (2009).

18. Kim, T., Hwang, W., Lee, H. & Kamm, R. D. Computational Analysis of Viscoelastic Properties of Crosslinked Actin Networks. *PLoS Computational Biology* **5**, e1000439 (2009).

19. Roeder, B. A., Kokini, K., Sturgis, J. E., Robinson, J. P. & Voytik-Harbin, S. L. Tensile Mechanical Properties of Three-Dimensional Type I Collagen Extracellular Matrices With Varied Microstructure. *J Biomech Eng* **124**, 214–222 (2002).

20. Xu, B., Chow, M.-J. & Zhang, Y. Experimental and Modeling Study of Collagen Scaffolds with the Effects of Crosslinking and Fiber Alignment. *International Journal of Biomaterials* **2011**, 1–12 (2011).

21. Chandran, P. L., Paik, D. C. & Holmes, J. W. Structural Mechanism for Alteration of Collagen Gel Mechanics by Glutaraldehyde Crosslinking. *Connective Tissue Research* **53**, 285–297 (2012).

22. Janmey, P. A. *et al.* Negative normal stress in semiflexible biopolymer gels. *Nature Materials* **6**, 48–51 (2007).

23. Motte, S. & Kaufman, L. J. Strain stiffening in collagen I networks. *Biopolymers* **99**, 35–46 (2013).

24. Munster, S. *et al.* Strain history dependence of the nonlinear stress response of fibrin and collagen networks. *Proceedings of the National Academy of Sciences* **110**, 12197–12202 (2013).

25. Roy, R., Boskey, A. & Bonassar, L. J. Processing of type I collagen gels using nonenzymatic glycation. *Journal of Biomedical Materials Research Part A* **9999A**, NA-NA (2009).

26. Mason, B. N., Starchenko, A., Williams, R. M., Bonassar, L. J. & Reinhart-King, C. A. Tuning three-dimensional collagen matrix stiffness independently of collagen concentration modulates endothelial cell behavior. *Acta Biomaterialia* **9**, 4635–4644 (2013).

27. Bordeleau, F. *et al.* Matrix stiffening promotes a tumor vasculature phenotype. *Proceedings of the National Academy of Sciences* **114**, 492–497 (2017).

28. Kim, O. V. *et al.* Compression-induced structural and mechanical changes of fibrin-collagen composites. *Matrix Biology* **60–61**, 141–156 (2017).

29. Kim, O. V., Litvinov, R. I., Weisel, J. W. & Alber, M. S. Structural basis for the nonlinear mechanics of fibrin networks under compression. *Biomaterials* **35**, 6739–6749 (2014).

30. Kim, O. V. *et al.* Foam-like compression behavior of fibrin networks. *Biomechanics and Modeling in Mechanobiology* **15**, 213–228 (2016).

31. Ramtani, S., Takahashi-Iñiguez, Y., Helary, C., Geiger, D. & Guille, M. M. G. Mechanical behavior under unconfined compression loadings of dense fibrillar collagen matrices mimetic of living tissues. *Journal of Mechanics in Medicine and Biology* **10**, 35–55 (2010).

32. Lane, B. A. *et al.* Constitutive modeling of compressible type-I collagen hydrogels. *Medical Engineering & Physics* **53**, 39–48 (2018).

33. Ban, E. *et al.* Strong triaxial coupling and anomalous Poisson effect in collagen networks. *PNAS* 201815659 (2019). doi:10.1073/pnas.1815659116

34. Biot, M. A. Mechanics of Deformation and Acoustic Propagation in Porous Media. *Journal of Applied Physics* **33**, 1482–1498 (1962).

35. Truesdell, C. & Toupin, R. The Classical Field Theories. in *Principles of Classical Mechanics and Field Theory / Prinzipien der Klassischen Mechanik und Feldtheorie* (ed. Flügge, S.) 226–858 (Springer Berlin Heidelberg, 1960). doi:10.1007/978-3-642-45943-6_2

36. Busby, G. A., Grant, M. H., MacKay, S. P. & Riches, P. E. Confined compression of collagen hydrogels. *Journal of Biomechanics* **46**, 837–840 (2013).

37. Voytik-Harbin, S. L., Roeder, B. A., Sturgis, J. E., Kokini, K. & Robinson, J. P. Simultaneous Mechanical Loading and Confocal Reflection Microscopy for Three-Dimensional Microbiomechanical Analysis of Biomaterials and Tissue Constructs. *Microscopy and Microanalysis* **9**, 74–85 (2003).

38. Bachrach, N. M., Mow, V. C. & Guilak, F. Incompressibility of the solid matrix of articular cartilage under high hydrostatic pressures. *Journal of Biomechanics* **31**, 445–451 (1998).
39. Chandran, P. L. & Barocas, V. H. Microstructural Mechanics of Collagen Gels in Confined Compression: Poroelasticity, Viscoelasticity, and Collapse. *Journal of Biomechanical Engineering* **126**, 152 (2004).
40. Knapp, D. M. *et al.* Rheology of reconstituted type I collagen gel in confined compression. *Journal of Rheology* **41**, 971–993 (1997).
41. Girton, T. S., Barocas, V. H. & Tranquillo, R. T. Confined Compression of a Tissue-Equivalent: Collagen Fibril and Cell Alignment in Response to Anisotropic Strain. *Journal of Biomechanical Engineering* **124**, 568 (2002).
42. Mow, V. C., Kuei, S. C., Lai, W. M. & Armstrong, C. G. Biphasic Creep and Stress Relaxation of Articular Cartilage in Compression: Theory and Experiments. *J Biomech Eng* **102**, 73–84 (1980).
43. Stein, A. M., Vader, D. A., Weitz, D. A. & Sander, L. M. The micromechanics of three-dimensional collagen-I gels. *Complexity* **16**, 22–28 (2011).
44. Licup, A. J. *et al.* Stress controls the mechanics of collagen networks. *Proceedings of the National Academy of Sciences* **112**, 9573–9578 (2015).
45. Yang, L. *et al.* Micromechanical bending of single collagen fibrils using atomic force microscopy. *Journal of Biomedical Materials Research Part A* **82A**, 160–168 (2007).
46. Yang, L. *et al.* Mechanical Properties of Native and Cross-linked Type I Collagen Fibrils. *Biophysical Journal* **94**, 2204–2211 (2008).

47. Yang, L., van der Werf, K. O., Dijkstra, P. J., Feijen, J. & Bennink, M. L. Micromechanical analysis of native and cross-linked collagen type I fibrils supports the existence of microfibrils. *Journal of the Mechanical Behavior of Biomedical Materials* **6**, 148–158 (2012).

SUPPLEMENTARY FIGURES

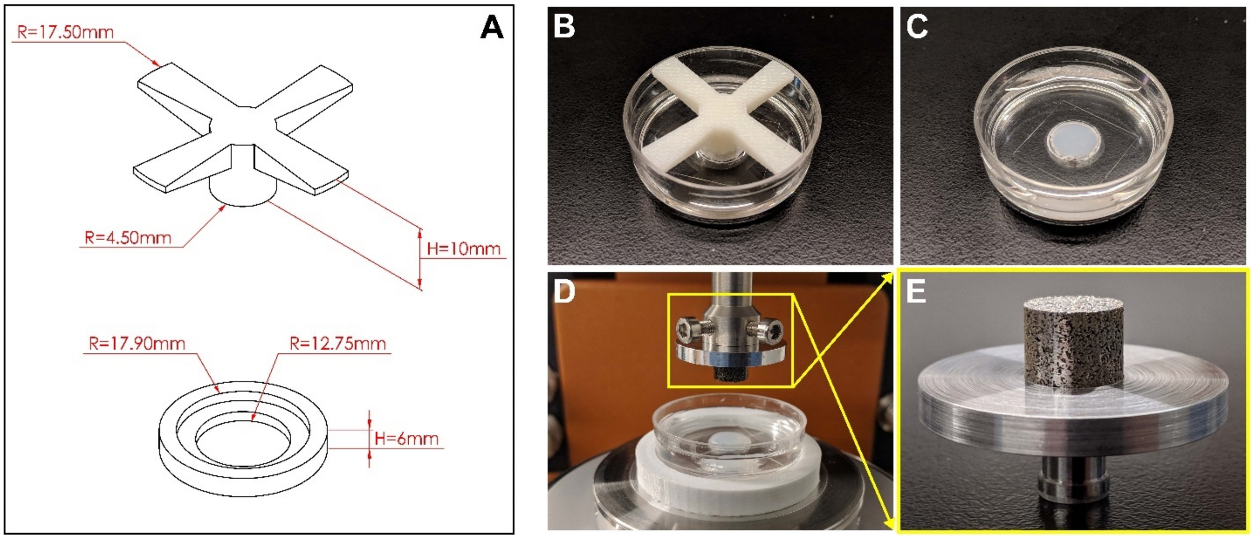

Supplementary Figure 1. Components needed to perform confined compression experiments on collagen hydrogels. 3D-printed custom parts (A) include a self-centering cylinder (top) and a centering ring (bottom). The self-centering cylinder was used to generate 9 mm PDMS wells in the middle of commercial 35 mm glass-bottom Petri dishes (B). The PDMS wells were in turn used to generate cylindrical collagen hydrogels (C) which were tested mechanically using a DHR-2 rheometer (D). An 8 mm porous indenter (E) was press-fitted within a 25 mm disposable plate to allow interstitial fluid flow during compression. Centering of the indenter with the respect to the PDMS well (used to confine collagen deformations) was guaranteed by the use of the custom centering ring.

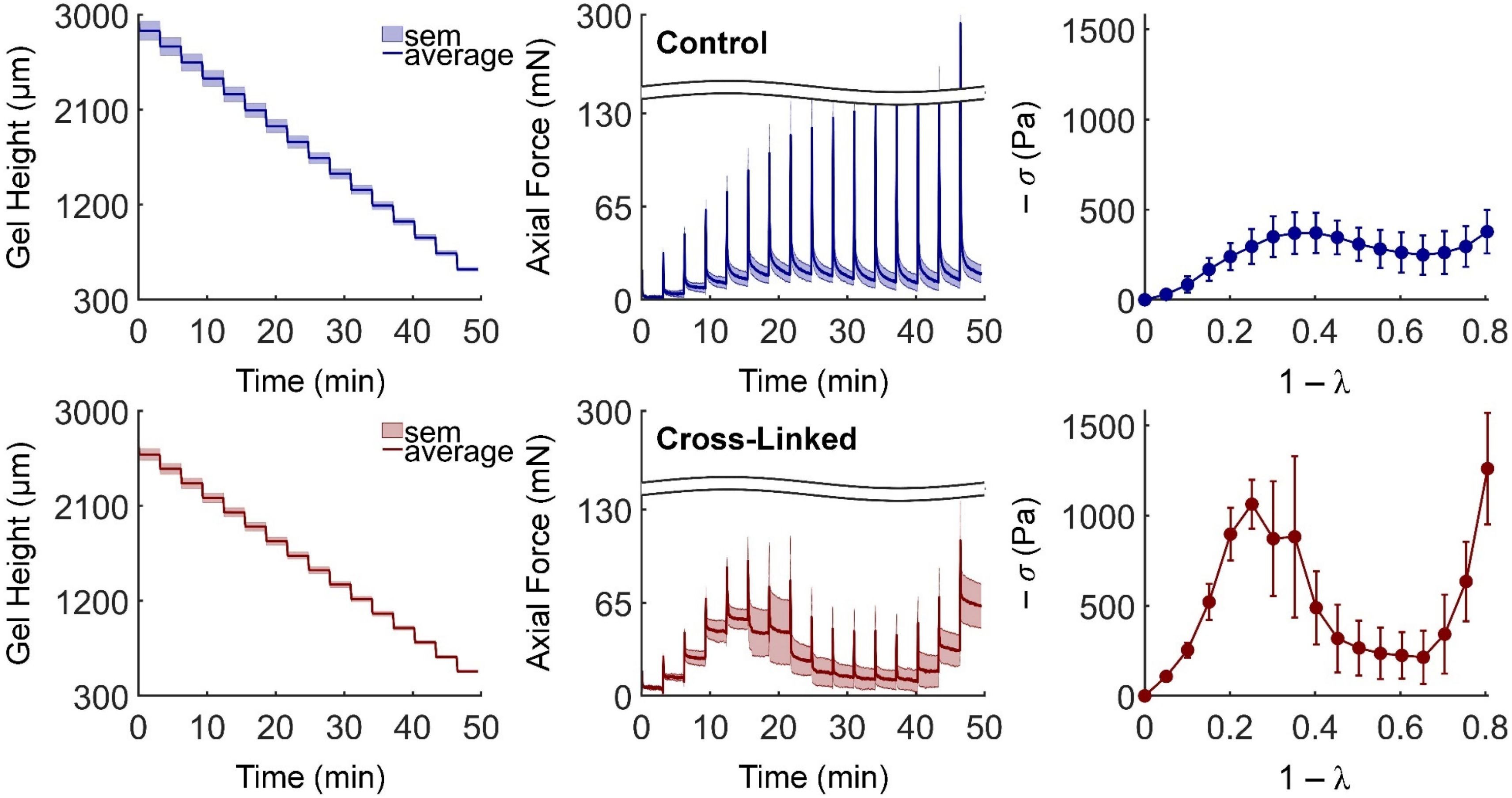

Supplementary Figure 2. Large deformation behavior of 4 mg/mL collagen gels. Control (top row) and cross-linked (bottom row) gels were compressed up to 80% of their original height by means of 5% steps (left column). Control gels developed higher peak forces followed by a decay to lower equilibrium forces with respect to cross-linked gels (middle column). On the other hand, cross-linked gels developed significantly higher equilibrium stresses (right column). It should be noted that, while control gels built up stresses smoothly

and reached a plateau at large deformations, cross-linked gels underwent frank rupture above ~20% compression. This finding informed subsequent tests that were conducted up to 18% compression by means of 3% steps. It should be noted that the plateau is followed by a further increase in stress, likely due to extreme compaction of collagen.

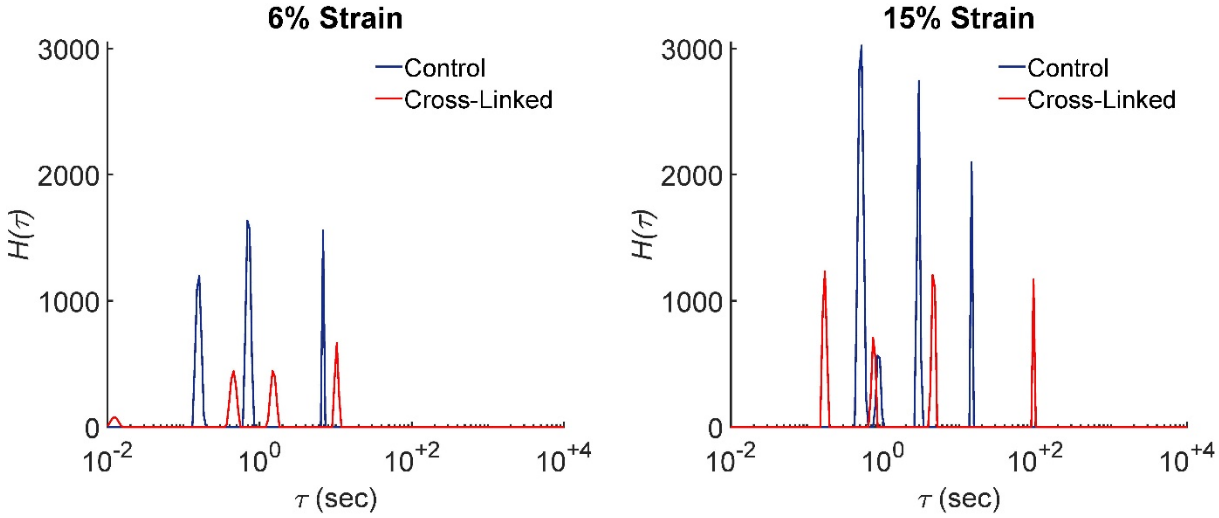

Supplementary Figure 3. Relaxation time spectra of representative 4 mg/mL control (blue lines) and cross-linked (red lines) gels exposed to 6% strain (left) and 15% strain (right). The relaxation time spectra are characterized by a finite series of discrete peaks, each associated with a distinct relaxation time constant. In this study we found that collagen gels exhibit consistently 3 to 4 peaks, with the area under each peaks – indicative of the energy dissipated by the respective relaxation mechanism – increasing for higher compressive strains. In addition, the area under the peaks was consistently lower for cross-linked, with respect to control, gels at each strain level.

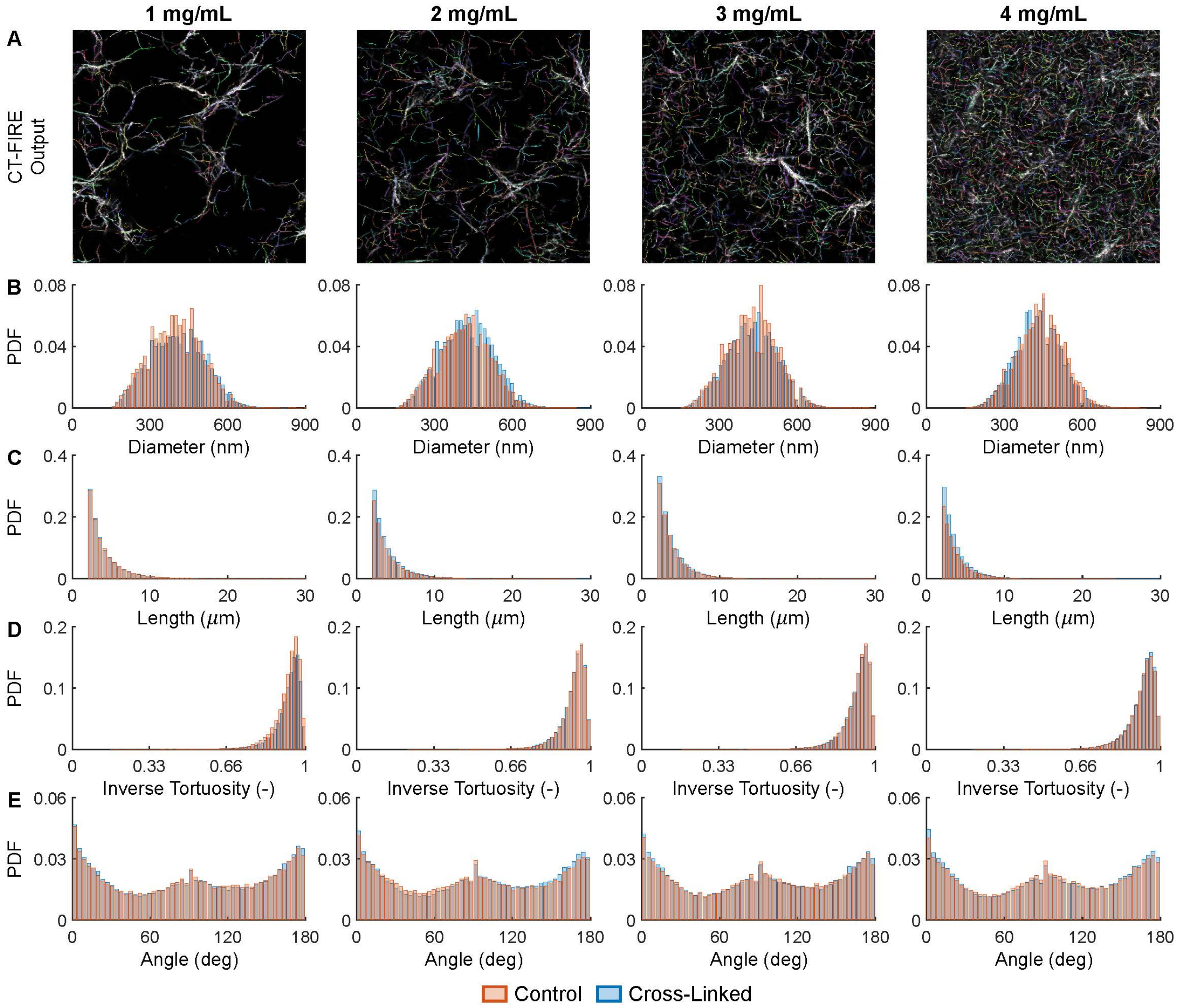

Supplementary Figure 4. Output from CT-FIRE analysis of control and cross-linked gels at all concentrations. (A) Representative images segmented via CT-FIRE with different colors indicating different fibers. Typical outputs include B) fiber diameter (or width), (C) fiber length, (D) inverse tortuosity (or straightness), and (E) fiber angle. The normalized histograms shown herein were obtained by pooling together data from all samples presented in Figure 4 and Supplementary Table 1. The distribution of inverse tortuosity is heavily skewed towards unity, which suggests fairly straight fibers. It should also be noted that all reconstituted collagen networks are characterized by a nearly random distribution of fiber angles. Such randomness in fiber

alignment underlies the assumption of isotropy in both material and fluid transport properties made in this study.

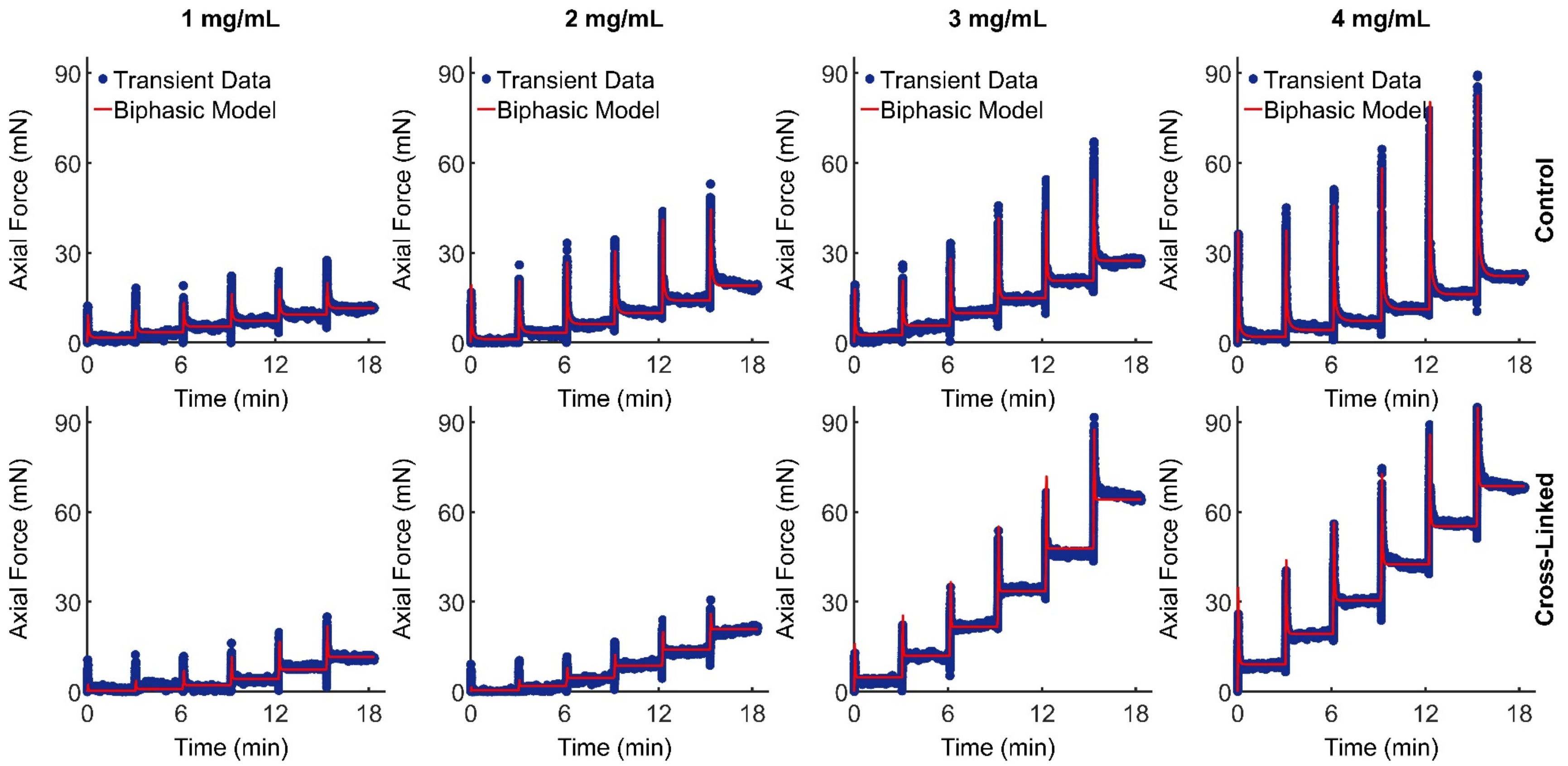

Supplementary Figure 5. Representative transient responses for control (top row) and cross-linked (bottom row) collagen gels at various concentrations (1-4 mg/mL). Experimental data are shown as blue circles and theoretical fits to a biphasic mixture model are shown as red lines. Nonlinear least squares fitting of equilibrium and transient responses led, respectively, to the determination of material parameters and hydraulic permeabilities (c.f., Supplementary Table 2).

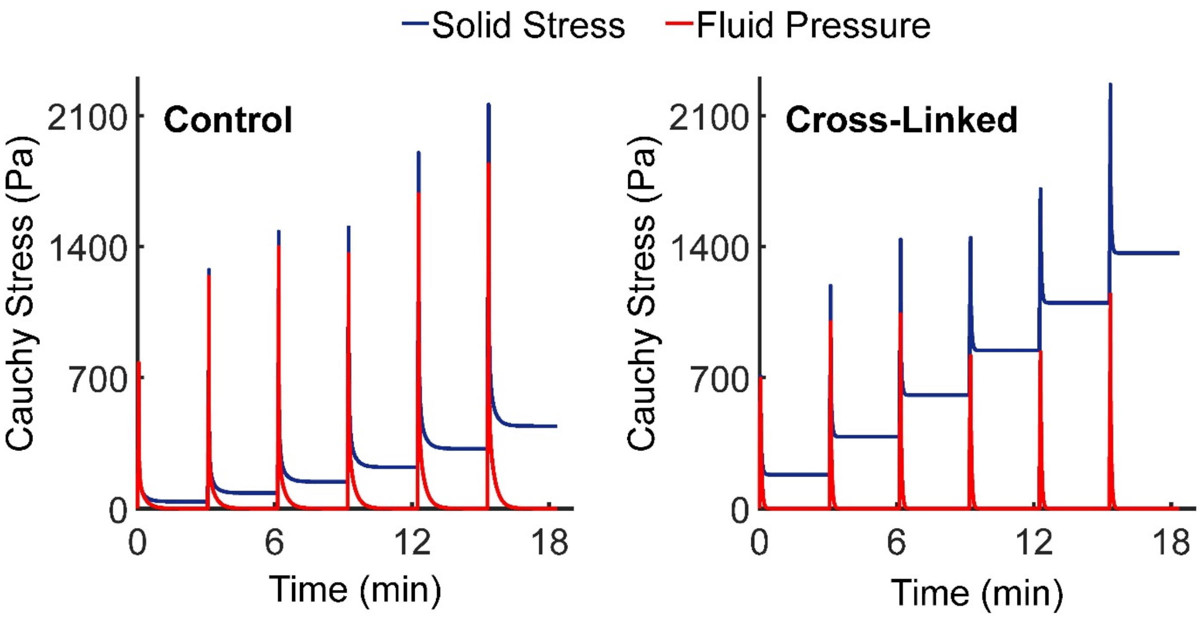

Supplementary Figure 6. Biphasic modeling allows estimation of solid stress (blue lines) and fluid pressure (red lines) generated by compression of representative control (left) and cross-linked (right) collagen gels. It should be noted how, after cross-linking, the solid stress increases due to material stiffening while the fluid pressure decays more rapidly due to lack of localized plastic remodeling.

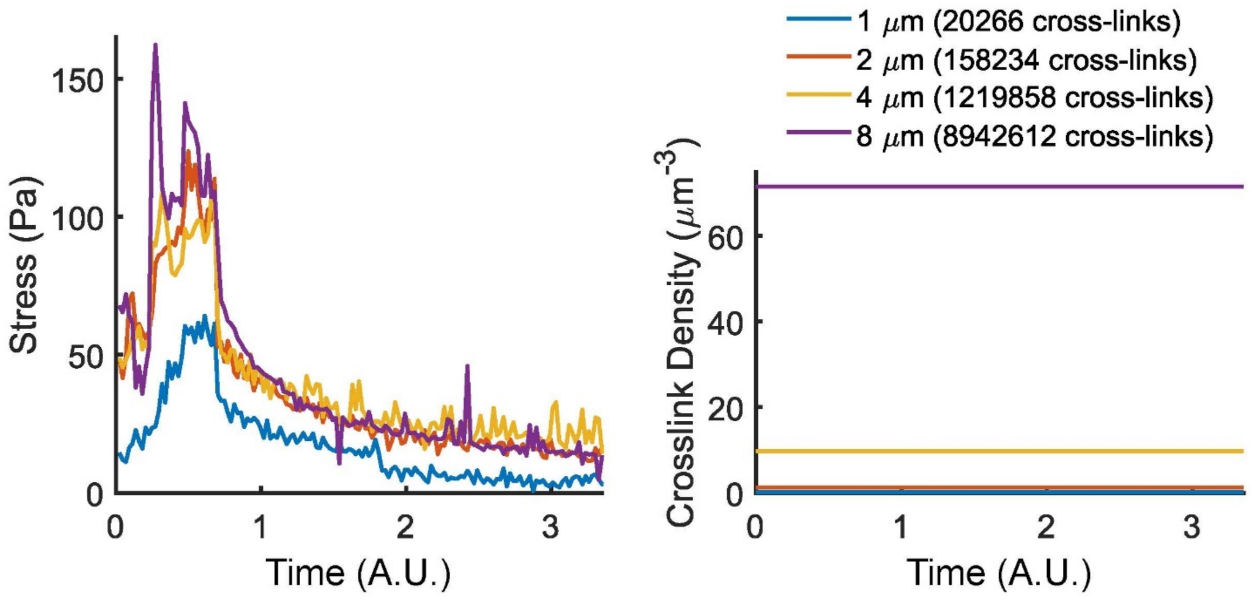

Supplementary Figure 7. Role played by the cross-linking distance in determining the stress response (left) and cross-link density (right) in a simulated fiber network under compression. The cross-linking distance represents the maximum distance within which the nodes form a covalent cross-link. Therefore, by increasing the cross-linking distance the number of cross-links increases, and this slows down significantly the computational time. We show that the stress generated in the network increases if we change the cross-linking distance from 1 μm to 2 μm, while it is insensitive to further changes despite they lead to a dramatic increase in the number of cross-links.

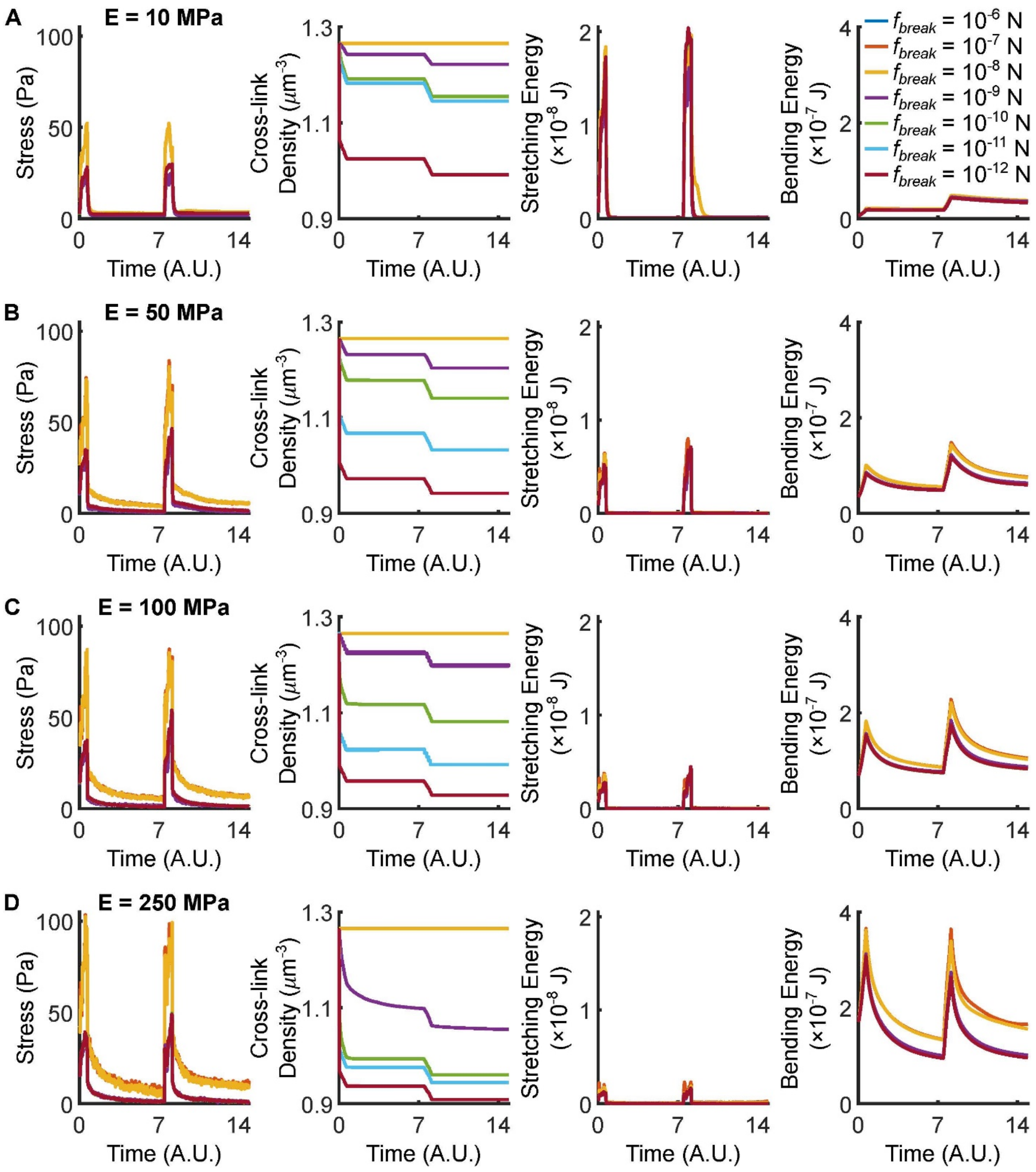

Supplementary Figure 8. Parametric analysis of simulated network responses to two consecutive steps of compression. The key parameters controlling network mechanics are the Young's modulus E of individual fibers and the cross-link breaking force f_{break}. Starting from E = 10 MPa (A), we increased the Young's modulus 5-fold to E = 50 MPa (B), 10-fold to E = 100 MPa (C), and 25-

fold to $E = 250$ MPa (D). For each value of E, we varied f_{break} over a broad range of values ranging between 1 pN (10^{-12} N) to 1 μN (10^{-6} N). All the other parameters are maintained equal. We show the evolution of the simulated stress at the compression boundary (first column), cross-link density (second column), stretching energy (third column), and bending energy (fourth column). The network responses simulated for low values of E and/or f_{break} are reminiscent of the mechanical behavior of control gels, with negligible equilibrium stresses developed after multiple steps of compression. Increasing E causes an increase in the bending energy and a simultaneous decrease in the stretching energy stored in the fibers. Increasing f_{break}, instead, leads to a gradual decrease in cross-link rupture events, as shown from the cross-link density plots, thus allowing mechanical loads to be transmitted to other fibers below the compression boundary. Interestingly, when increasing f_{break} from 1 nN (10^{-9} N) to 10 nN (10^{-8} N) and above, the cross-link density remains constant and the network stiffens, as shown by the higher values of peak and equilibrium stress obtained from our simulations. The network stiffening observed increasing f_{break} is more marked at higher values of E, thus showing that both fiber stiffening and cross-link strengthening are necessary to simulate the stiffer mechanical response observed experimentally upon GA cross-linking.

SUPPLEMENTARY TABLES

Supplementary Table 1. Microstructural features of collagen networks as a function of concentration (1-4 mg/mL) and cross-linking.

**Control**	Fiber Density (μm^{-3})	Fiber Diameter (nm)	Fiber Length (μm)	Matrix Porosity (-)	Autofluorescence (A.U.)
1 mg/mL	0.063 ± 0.003	406.1 ± 2.0	4.09 ± 0.02	0.922 ± 0.006	68 ± 2
2 mg/mL	0.117 ± 0.010†	422.4 ± 3.8	3.99 ± 0.02	0.817 ± 0.022†	82 ± 3
3 mg/mL	0.146 ± 0.003†‡	425.6 ± 3.7†	3.82 ± 0.04†‡	0.801 ± 0.022†	91 ± 7†
4 mg/mL	0.185 ± 0.005†‡⁑	428.9 ± 4.8†	3.68 ± 0.04†‡	0.803 ± 0.021†	95 ± 6†
**Cross-Linked**	Fiber Density (μm^{-3})	Fiber Diameter (nm)	Fiber Length (μm)	Matrix Porosity (-)	Autofluorescence (A.U.)
1 mg/mL	0.077 ± 0.001*	397.8 ± 0.6*	4.10 ± 0.02	0.918 ± 0.002	377 ± 18*
2 mg/mL	0.101 ± 0.003†	414.1 ± 0.5†	4.00 ± 0.01†	0.870 ± 0.004†	519 ± 29†*
3 mg/mL	0.143 ± 0.008†‡	424.5 ± 1.5†‡	3.83 ± 0.03†‡	0.820 ± 0.007†‡	650 ± 27†‡*
4 mg/mL	0.192 ± 0.001†‡⁑	440.3 ± 1.0†‡⁑	3.71 ± 0.01†‡⁑	0.807 ± 0.005†‡	633 ± 7†‡*

†p<0.05 with respect to 1 mg/mL

‡p<0.05 with respect to 2 mg/mL

⁑p<0.05 with respect to 3 mg/mL

*p<0.05 with respect to Control

Supplementary Table 2. Biomechanical features of collagen networks as a function of concentration (1-4 mg/mL) and cross-linking. Best-fit material parameters of the Yeoh model and the isotropic, strain-independent hydraulic permeability were determined via nonlinear regression. Goodness of fit was measured by the coefficient of determination R^2.

**Control**	Material Parameters				Hydraulic Permeability	
	c_1 (Pa)	c_2 (Pa)	c_3 (Pa)	R^2	k ($\times 10^{-10}$ m^4/Ns)	R^2
1 mg/mL	323 ± 139	-193 ± 143	39 ± 39	0.819 ± 0.161	0.461 ± 0.148	0.767 ± 0.162
2 mg/mL	159 ± 89	-318 ± 192	374 ± 164	0.964 ± 0.018	0.088 ± 0.031†	0.902 ± 0.024
3 mg/mL	201 ± 106	-465 ± 209	636 ± 228	0.663 ± 0.207	0.034 ± 0.009†	0.897 ± 0.021
4 mg/mL	229 ± 148	-231 ± 110	366 ± 247	0.723 ± 0.211	0.037 ± 0.015†	0.840 ± 0.063
**Cross-Linked**	Material Parameters				Hydraulic Permeability	
	c_1 (Pa)	c_2 (Pa)	c_3 (Pa)	R^2	k ($\times 10^{-10}$ m^4/Ns)	R^2
1 mg/mL	306 ± 88	-182 ± 109	278 ± 155	0.968 ± 0.012	1.173 ± 0.283*	0.909 ± 0.025
2 mg/mL	403 ± 109	-193 ± 127	326 ± 184	0.961 ± 0.014	0.358 ± 0.123†	0.880 ± 0.038
3 mg/mL	724 ± 144*	-869 ± 340	1474 ± 360	0.996 ± 0.001	0.092 ± 0.020†*	0.985 ± 0.002
4 mg/mL	867 ± 212*	-542 ± 193	1598 ± 516	0.997 ± 0.001	0.085 ± 0.022†	0.987 ± 0.002

†p<0.05 with respect to 1 mg/mL

*p<0.05 with respect to Control

Supplementary Table 3. Collagen gel thickness after confined compression testing measured from SHG images acquired using a 16x objective. All gels were 3 mm thick prior to compression and were allowed to recover overnight prior to thickness measurements.

**Gel Thickness (µm)**	Control	Cross-Linked
1 mg/mL	2.51 ± 0.03	2.80 ± 0.02*
2 mg/mL	2.60 ± 0.02†	2.78 ± 0.03*
3 mg/mL	2.70 ± 0.02†‡	2.85 ± 0.04*
4 mg/mL	2.74 ± 0.02†‡	3.02 ± 0.02†‡⁑*

†$p<0.05$ with respect to 1 mg/mL

‡$p<0.05$ with respect to 2 mg/mL

⁑$p<0.05$ with respect to 3 mg/mL

*$p<0.05$ with respect to Control

Supplementary Table 4. Main parameters used for the implementation of the discrete fiber network model. For each parameter, its name, value, and eventual source are listed. For a number of parameters, a range of values is provided either because the actual value is not known or because it was varied parametrically to establish its effect on the mechanical response of the 3D fiber network.

**Parameter**	**Value**	**Source**
Computational domain size	50 × 50 × 50 μm^3	–
Thickness for stress calculation	1 μm	–
Compression cycles	1-3	–
Compression step	3%	–
Compression rate	1-20 %/s	–
Period of static hold	3 s	–
Time step (dt)	10^{-4} s	–
Interstitial fluid viscosity (η)	10^5 cP	–
True mass density of collagen (ρ_T^c)	1.35 g/cm^3	[16]
Fiber diameter (d)	155 μm	[14,15]
Fiber length distribution ($PDF(l)$)		Equation (S13) / Figure S4
a	0.972	–
b	1.748 μm	–
Length of discretized segments (l_0)	1 μm	[15]
Fiber stiffness (E)	10-250 MPa	[14,45–47]
Cross-link distance (l_{xlink})	1-8 μm	–
Cross-link breaking force (f_{break})	10^{-12}-10^{-6} N	–

SUPPLEMENTARY VIDEOS

Supplementary Video 1. DIC time-lapse movie (48 hours) of a MCF-10A tumor spheroid embedded in 4 mg/mL collagen. Cancer cell proliferation and overall spheroid growth results in compression of the surrounding collagen.

Supplementary Video 2. Simulation of a stress relaxation test on a 4 mg/mL collagen network compressed to 15% strain. Fibers buckle under compression resulting in a localized densification of collagen near the compressed surface.
